# Targeting Emerging RNA Viruses by Engineered Human Superantibody to Hepatitis C Virus RNA-Dependent RNA Polymerase

**DOI:** 10.3389/fmicb.2022.926929

**Published:** 2022-07-22

**Authors:** Kittirat Glab-ampai, Kanasap Kaewchim, Techit Thavorasak, Thanatsaran Saenlom, Watayagorn Thepsawat, Kodchakorn Mahasongkram, Kanyarat Thueng-In, Nitat Sookrung, Wanpen Chaicumpa, Monrat Chulanetra

**Affiliations:** ^1^Center of Research Excellence in Therapeutic Proteins and Antibody Engineering, Department of Parasitology, Faculty of Medicine Siriraj Hospital, Mahidol University, Bangkok, Thailand; ^2^Graduate Program in Immunology, Department of Immunology, Faculty of Medicine Siriraj Hospital, Mahidol University, Bangkok, Thailand; ^3^School of Pathology, Translational Medicine Program, Institute of Medicine, Suranaree University of Technology, Nakhon Ratchasima, Thailand; ^4^Biomedical Research Incubator Unit, Department of Research, Faculty of Medicine Siriraj Hospital, Mahidol University, Bangkok, Thailand

**Keywords:** RNA viruses, RNA-dependent RNA polymerase, phage display, human single-chain antibody variable fragment, superantibody (cell penetrating antibody), computerized simulation, plaque-forming assay, focus-forming assay

## Abstract

RNA-dependent RNA polymerase (RdRp) is a unique and highly conserved enzyme across all members of the RNA virus superfamilies. Besides, humans do not have a homolog of this protein. Therefore, the RdRp is an attractive target for a broadly effective therapeutic agent against RNA viruses. In this study, a formerly generated cell-penetrating human single-chain antibody variable fragment (superantibody) to a conformational epitope of hepatitis C virus (HCV) RdRp, which inhibited the polymerase activity leading to the HCV replication inhibition and the host innate immunity restoration, was tested against emerging/reemerging RNA viruses. The superantibody could inhibit the replication of the other members of the *Flaviviridae* (DENV serotypes 1−4, ZIKV, and JEV), *Picornaviridae* (genus *Enterovirus*: EV71, CVA16), and *Coronaviridae* (genus *Alphacoronavirus*: PEDV, and genus *Betacoronavirus*: SARS-CoV-2 (Wuhan wild-type and the variants of concern), in a dose-dependent manner, as demonstrated by the reduction of intracellular viral RNAs and numbers of the released infectious particles. Computerized simulation indicated that the superantibody formed contact interfaces with many residues at the back of the thumb domain (thumb II site, T2) of DENV, ZIKV, JEV, EV71, and CVA16 and fingers and thumb domains of the HCV and coronaviruses (PEDV and SARS-CoV-2). The superantibody binding may cause allosteric change in the spatial conformation of the enzyme and disrupt the catalytic activity, leading to replication inhibition. Although the speculated molecular mechanism of the superantibody needs experimental support, existing data indicate that the superantibody has high potential as a non-chemical broadly effective anti-positive sense-RNA virus agent.

## Introduction

During the past two decades, several human and animal RNA viruses have emerged/reemerged to cause epidemics/epizootics or pandemics/panzootics that inflict a huge negative impact on the human and animal health as well as socioeconomics globally. Examples are influenza A viruses (IAV H5N1 and H1N1pdm2009) (Tang et al., 1998; Novel Influenza A/H1N1 Investigation Team, 2009); flaviviruses including dengue virus (DENV) (Kyle and Harris, 2008; European Centre for Disease Prevention and Control, 2020) and zika virus (ZIKV) (Noobrakhsh et al., 2019); ebola virus (EBOV) (World Health Organization Ebola Response Team, 2014); enteroviruses including EV71 and CVA16 (Schmidt et al., 1974); and coronaviruses (CoVs) including *Alphacoronavirus* (porcine epidemic diarrhea virus, PEDV), *Betacoronavirus* (severe acute respiratory syndrome virus, SARS-CoV; MERS-CoV; novel coronavirus 2019 or SARS-CoV-2), and *Deltacoronavirus* (porcine *Deltacoronavirus*, PDCoV) (Pensaert and de Bouck, 1978; Chan-Yeng et al., 2015; Hu et al., 2015; Jung et al., 2016; World Health Organization [WHO], 2019). Currently, the world population is facing the unprecedentedly scaled pandemic of coronavirus disease caused by the SARS-CoV-2, named COVID-19, that emerged in December 2019. The catastrophic COVID-19 pandemic caused by the SARS-CoV-2 mutated descendants (variants of concern, VOC) is still going on, although a large fraction of the world population has been vaccinated against the disease. As of March 10, 2022, more than 400 million people around the globe were infected by the SARS-CoV-2, and among them, more than 6 million were deceased. The world consternations frequently threatened by the emerging/re-emerging RNA viruses emphasize the need not only for effective vaccines but also for safe therapeutics to counteract the viruses, especially for those with severe morbidity.

RNA-dependent RNA polymerase (RdRp) is a highly conserved enzyme across all members of the RNA virus superfamilies (except *Retroviridae*), although the enzyme itself accounts for the rapid RNA virus mutations from the high rate of transcription errors. The RNA virus RdRps probably arose from a common ancestor (Payne, 2017). The enzyme is indispensable for the synthesis of the genomic RNA and the transcription process during the virus replication cycle (Payne, 2017). Positive-sense RNA viruses use their RNA genomes as mRNAs for protein synthesis, while the negative-sense RNA viruses use the genomic RNAs as templates of the RdRp-dependent transcriptional process in the generation of the plus-sense strand that functions as mRNAs. Some RNA viruses, including coronaviruses, use RdRp for subgenomic RNA synthesis. Although the RdRps of the RNA viruses are diverse in their amino acid sequences as well as the structural details (the RdRp molecule may be linked with other structures that perform other functions, such as methyltransferase, endonuclease, helicase, and nucleoside-triphosphatase), their catalytic modules are relatively conserved and composed of the palm, fingers, and thumb domains such that the overall architecture reminisces encircled/cupped human right hand (Jia and Gong, 2019). The catalytic motif (active site) of the RdRp is surrounded by the palm, fingers, and thumb domains with seven catalytic motifs (motifs A–G) distributed within the palm (motifs A–E) and fingers (motifs F–G) (Poch et al., 1989; Gorbalenya et al., 2002; Bruenn, 2003; te Velthuis, 2014; Wu et al., 2015; Venkataraman et al., 2018; Jia and Gong, 2019). The viral RdRp lacks a human homolog. It is the essential and most conserved protein of RNA viruses (Jia and Gong, 2019). The RdRps of *Flaviviridae*, Hepatitis C virus (HCV), DENV, ZIKV, West Nile virus, share high percentages of identity with RdRp of the *Coronaviridae* members (e.g., SARS-CoV, MERS-CoV, and SARS-CoV-2) (Picarazzi et al., 2020); it is highly plausible that drugs or therapeutics that act on the RdRp of the former virus family may as well affect the RdRp of the latter, if not also other families. This speculation is well supported by the evidence that sofosbuvir (a small molecular inhibitor of HCV RdRp/NS5B protein in combination with daclatasvir/Daklinza) showed effectiveness in reducing the mortality rate of patients with severe COVID-19 (Abbass et al., 2021; Zein et al., 2021). In this study, therefore, we tested a previously generated cell-penetrating human single-chain antibody (superantibody) to HCV RdRp that has been shown to effectively interfere with the HCV replication and rescued the virally suppressed host innate immunity (Thueng-In et al., 2014), for replication inhibition of several other positive-sense RNA viruses. The ultimate purpose is to develop the broadly effective superantibody further toward a clinical use as a pan, direct-acting anti-positive-sense RNA virus agent.

## Materials and Methods

### Cells, Viruses, and Virus Propagation

Human hepatocellular carcinoma cells (Huh7), human embryonic kidney (HEK) 293T cells, African green monkey kidney epithelial (Vero) cells, and Rhabdomyosarcoma (RD) cells were obtained from American Type Culture Collection (ATCC, Manassas, VA, United States). Vero E6 cells were provided by Prasert Auewarakul, Department of Microbiology, Faculty of Medicine Siriraj Hospital, Mahidol University, Bangkok. Cells were cultured in Dulbecco’s modified Eagle’s medium (DMEM) (Gibco, Thermo Fisher Scientific, Waltham, MA, United States) supplemented with 10% fetal bovine serum (FBS) (HyClone; GE Healthcare Life Sciences, Marlborough, MA, United States), 100 units/mL penicillin, 100 mg/mL streptomycin, and 2 mM L-glutamine (Gibco) (complete DMEM).

The viruses used in this study included HCV infectious particles, one isolate each of DENV serotypes 1-4; one isolate of ZIKV; one isolate of Japanese encephalitis virus (JEV); one isolate each of Wuhan wild-type, alpha (B.1.1.7), beta (B.1.351), delta (B.1.617.2), and omicron (B.1.1.529) of SARS-CoV-2; Enterovirus 71 (EV71, genotype A, BrCr strain, ATCCR-VR-1775TM); and Coxsackievirus A16 (CVA16) and PEDV (P70 strain, GII field isolate from a deceased infected piglet in Thailand).

The HCV infectious particles were prepared as described previously (Thueng-In et al., 2014). Full-length cDNA of pJFH-1 (Wakita et al., 2005) was linearized by digesting the plasmid with *Xba*I endonuclease (Fermentas, Burlington, ON, Canada), and 1 μg was transcribed *in vitro* by using a Megascript T7 kit (Ambion, Carlsbad, CA, United States). The RNA transcript (10 μg) was electroporated into Huh7 cells (4.0 × 10^6^ cells) in 0.4 mL of the serum reduced medium (Opti-MEM) (Invitrogen, Thermo Fisher Scientific) by using a single pulse at 0.27 kV and 100 milli-s. The transfected cells were immediately transferred to 40 mL of complete DMEM and seeded into wells of a 12-well culture plate (2 × 10^5^ cells/well). The plate was incubated at 37°C in a 5% CO_2_ atmosphere for 5 days. The culture supernatant containing the HCV infectious particles was concentrated by using a centrifugal device (Pall, Port Washington, NY, United States). The virus titer was determined by focus-forming assay (FFA).

DENV (serotypes 1-4), ZIKV, and JEV were propagated in Vero cells maintained in complete DMEM at 37°C in a 5% CO_2_ incubator for 3−5 days. The culture supernatants containing the viruses were collected, and the virus titers (pfu/mL) were determined by the plaque-forming assay (PFA). The viruses were kept in small portions at −80°C as the stocks.

The enteroviruses (EV71 and CVA16) were propagated in RD cells grown in complete DMEM at 37°C in a 5% CO_2_ atmosphere (Phanthong et al., 2020). The cytopathic effect (CPE) characterized by cell rounding, clumping, and/or detaching was observed daily. The culture was harvested (both cells and spent medium) when the CPE was at maximum and subjected to three freeze-thaw cycles, centrifuged to remove the cell debris, and the supernatant containing the virus was kept in small aliquots at –80°C as the virus stocks. The cell culture infectious dose 50 (CCID_50_) of the virus stock was determined (Phanthong et al., 2020). Briefly, the virus stock was 10-fold serially diluted in complete DMEM and then added to the wells of 96-well culture plates. RD cells (2 × 10^4^ cells) were added to each virus-containing well; the plate was incubated at 37°C in a 5% CO_2_ atmosphere until the CPE was clearly observed. The Kärber formula (World Health Organization [WHO], 2004) was used to calculate the virus CCID_50_ (10*^x^*/mL) for each viral stock.

Porcine epidemic diarrhea virus (PEDV) was propagated in the permissive Vero cells as for the DENV, ZIKV, and JEV for 2 days. The amount of the PEDV in the harvested cell spent medium was determined by the plaque (syncytial)-forming assay (Thavorasak et al., 2022). The virus was kept at −80°C in small portions until use.

### Preparation of Cell-Penetrating Human Superantibody to Hepatitis C Virus RNA-Dependent RNA Polymerase

HB2151 *E. coli* clone 34 that carried pCANTAB5E phagemid with inserted gene sequence coding for human single-chain antibody variable fragment (*huscfv*) specific to HCV RdRp (HuscFv34) was generated previously by using phage display technology (Thueng-In et al., 2014). Recombinant HCV NS5BΔ55 (RdRp) protein was used as an antigen in the phage bio-panning to select out the antigen-bound phage clones from the HuscFv phage display library (Kulkeaw et al., 2009). One of the HB2151 *E. coli* clones (clone 34) infected with the antigen-bound phage produced HuscFv (HuscFv34) that inhibited the HCV RdRp activity *in vitro* (Thueng-In et al., 2014) and when the HuscFv34 was linked to a cell-penetrating peptide (CPP), i.e., penetratin (PEN), the PEN-HuscFv34 could enter the Huh7 cells (being superantibody). The superantibody not only inhibited HCV replication *ex vivo* but also rescued the host’s innate immunity from the HCV suppression (Thueng-In et al., 2014).

In this study, the *huscfv* from the recombinant *huscfv*-phagemid of the HB2151 *E. coli* clone 34 was subcloned to recombinant pET23b+ plasmid backbone carrying a DNA insert coding for a protein transduction domain/cell-penetrating peptide, penetratin (PEN) (Poungpair et al., 2010), and the DNA construct was introduced to BL21(DE3) *E. coli.* Non-chromatographic purification of the *E. coli* inclusion body (IB) was used to isolate the PEN-HuscFv34 from the bacterial cells grown under IPTG induction conditions. Four grams of the bacterial pellet was resuspended with 20 mL of 1 × BugBuster^®^ protein extraction reagent (Millipore, Merck KGaA, Darmstadt, Germany) dissolved in 50 mM tris(hydroxymethyl)aminomethane (Tris; Millipore, Merck KGaA), pH 8.0. After the bacterial pellet was completely resuspended, Lysonase™ bioprocessing reagent (Millipore, Merck, KGaA) was added at 10 μL per gram of the wet bacterial pellet. After 20 min at room temperature (25 ± 2°C) on a slow setting shaking platform, the soluble fraction was removed from the preparation by centrifugation at 8000 × *g* for 30 min. The insoluble contents was washed with wash-100 reagent [50 mM phosphate buffer, pH 8.0, 500 mM sodium chloride (Kemaus, CherryBrook, NSW, Australia), 5 mM ethylenediaminetetraacetic acid (Kemaus), 8% (*v/v*) glycerol (Kemaus), and 1% (*v/v*) Triton X-100 (USB, Affymetrix, Thermo Fisher Scientific)] at 25°C and wash-114 reagent [50 mM Tris-HCl pH 8.0, 500 mM sodium chloride, 1% (*v/v*) Triton X-114 (Sigma Aldrich, St. Louis, MO, United States)] at 4°C. The preparation was spun down at 8000 × *g* for 30 min and the supernatant was removed. The inclusion body was washed with deionized distilled water and collected by centrifugation at 8000 × *g* for 30 min. The PEN-HuscFv34 was retrieved from the inclusion body by solubilization and refolding. The inclusion body solubilization was performed by dissolving the inclusion body in 50 mM *N*-cyclohexyl-3-aminopropanesulfonic acid (CAPS) (Sigma Aldrich, St. Louis, MO, United States) buffer, pH 10.8 supplemented with 0.3% (*w/v*) sodium lauroyl sarcosinate (Sigma Aldrich, St. Louis, MO, United States) and 1 mM dithiothreitol (DTT; USB, Affymetrix) at a protein concentration of 1 mg/mL. Solvation of the inclusion body was performed at room temperature for 15 min followed by keeping at 4°C for 16 h. The non-solubilized part was removed by centrifugation at 10,000 × *g* for 10 min. The preparation was immediately refolded by buffer exchange against 20 mM imidazole, pH 8.5 with and without 0.1 mM DTT. The refolded PEN-HuscFv34 was subsequently verified by SDS-PAGE and Coomassie Brilliant Blue G-250 (CBB) staining.

### Verification of the Cell-Penetrating Ability of the Penetratin-HuscFv34

Human hepatocellular carcinoma cells (1 × 10^5^ cells) in complete DMEM were seeded onto a cover glass placed in a well of 24-well cell culture plate and incubated at 37°C in a 5% CO_2_ atmosphere overnight. The established cell monolayer was added with the PEN-HuscFv34 prepared from the *E. coli* inclusion body and kept at 37°C in a 5% CO_2_ atmosphere for 24 h. The cells were washed with PBS, fixed with 4% paraformaldehyde in PBS, and permeated with 0.1% Triton X-100 (USB, Affymetrix) in PBS. The cells were blocked with 5% BSA in PBS at room temperature for 20 min. After the excess BSA was removed by washing with PBS, rabbit anti-HuscFv34 was added to the cell monolayer and incubated for 1 h. Goat anti-rabbit Ig-AlexaFlour488 (1: 200; Thermo Fisher Scientific) was used as the secondary antibody, and DAPI was used to locate nuclei. After washing, the cells were mounted and observed under a confocal microscope (Nikon, Melville, NY, United States) for intracellular PEN-HuscFv34.

### Biocompatibility of the Penetratin-HuscFv34/Superantibody to Mammalian Cells

Mammalian cells including A549, Huh7, Vero, and Vero E6 cells (4 × 10^4^) were seeded separately in a 96-well white plate (Corning, Thermo Fisher Scientific) and incubated at 37°C in the CO_2_ incubator overnight. The fluids were discarded; the cells were replenished with a culture medium containing PEN-HuscFv34 (0.25, 0.5, 1.0, 1.5, and 2.0 μM) and kept at 37°C in the CO_2_ incubator overnight. Cytotoxicity of the superantibody was determined by using Cytotox-Glo™ Cytotoxicity Assay (Promega, Madison, WI, United States). The assay buffer provided with the kit was added to each well (50 μL/well), and the plate was kept at room temperature for 15 min. Experimental dead cell luminescence was detected by using Multidetection Microplate Reader Synergy H1 (Biotek, Agilent Technology, Santa Clara, CA, United States). Lysate reagent of the test kit was then added to all wells (50 μL/well), and the plate was placed on an orbital shaker (100 rpm) for 15 min. Total dead cell luminescence was detected, also by the microplate reader. Viable cell luminescence (Test luminescence) was calculated: Test luminescence = Total dead cell luminescence – Experimental dead cell luminescence. Percent cell viability was calculated: (Test luminescence ÷ Normal cell luminescence) × 100.

### RNA Virus Replication Inhibition Mediated by Superantibody

Ten micrograms of HCV-JFH1 RNA was transfected into Huh7 cells by electroporation. The transfected cells were immediately seeded to 12-well cell culture plate (2 × 10^5^ cells/well) and incubated at 37°C, 5% CO_2_ for 6 h. After washing the cells, the complete DMEM containing various concentrations of superantibody (0.25, 0.5, 1.0, 1.5, and 2.0 μM) or medium alone was added. The treated cells were cultured at 37°C in a 5% CO_2_ atmosphere for 5 days. The RNAs were extracted from the treated cells for viral RNA quantification by real-time PCR; the HCV infectious particles in the culture supernatants were enumerated by focus-forming assay (FFA).

Vero cells (3 × 10^5^ cells) were seeded to 12-well cell culture plates and incubated at 37°C in a 5% CO_2_ incubator overnight. DENV (serotypes 1-4), ZIKV, and JEV at MOI 0.1 and PEDV at MOI 0.0005 were added individually to the Vero cells in different wells and incubated further for 1 h. After washing with plain DMEM, complete DMEM containing the PEN-HuscFv34/superantibody (0.25, 0.5, 1.0, 1.5, and 2.0 μM) or medium alone (negative control) were added to appropriate wells containing the infected cells and incubated for 24 h for the PEDV and 48 h for the other viruses. The culture supernatants and cells were collected, and RNA was extracted from each cell sample and subjected to real-time RT-PCR for viral RNA quantification. Infectious virus particles in the culture supernatant samples were enumerated by the plaque-forming assay (PFA).

The RD cells were transfected with 100 CCID_50_/mL of EV71 strain BrCr or MOI 0.1 of CVA16. After 1 h incubation at 37°C in a 5% CO_2_ incubator, cells were washed one time with plain DMEM and replaced with complete DMEM containing superantibody (0.25, 0.5, 1.0, 1.5, and 2.0 μM) or medium alone as a negative control. Cells were incubated further for 24 h. Then, RNA was extracted from the collection and the viral RNA was quantified by real-time RT-PCR. Infectious virus particles in the culture supernatants were enumerated by PFA.

For SARS-CoV-2 replication inhibition, 1.5 × 10^5^ cells of Vero E6 cells were seeded to wells of 24-well cell culture plates and incubated at 37°C, 5% CO_2_ for 24 h. The plates were moved to the BSL-3 room to perform all the subsequent processes of the experiment. The seeded cells were infected with SARS-CoV-2 [Wuhan wild type, alpha (B.1.1.7), beta (B.1.351), delta (B.1.617.2), and omicron (B.1.1.529)] at 50 PFU/well. After 1 h incubation, the supernatants were removed and replenished with the superantibody (0.25, 0.5, 1.0, 1.5, and 2.0 μM) containing DMEM supplemented with the 2% FBS. The treated cells were incubated at 37°C, 5% CO_2_ for 18 h. The RNAs were extracted from the cells for the real-time RT-PCR, and the culture supernatants were collected to detect the infectious particles by PFA for the Wuhan wild type and α, β, and δ variants and by FFA for the omicron variant (their plaques in the PFA were too tiny to be counted accurately).

### Real-Time RT-PCR

The RNAs from the superantibody/medium-treated infected cells were extracted using TRIzol^®^ reagent (Invitrogen). The amounts of viral RNA were quantified by real-time RT-PCR using a 1-step brilliant III SYBR green RT-qPCR master mix (Agilent Technologies). The real-time RT-PCR primers for each virus and house-keeping gene control are listed in Supplementary Table 1. The copy numbers of viruses were calculated from the Cq value using a comparative method.

### Plaque-Forming Assay

The Vero or Vero E6 cells were seeded into wells of 24-well-culture plates (1.5 × 10^5^ cells per well) and kept in humidified 5% CO_2_ incubator at 37°C overnight. The virus-containing samples were diluted 10-fold serially, and 250 μL aliquots were added to the wells containing the cell monolayer. Experiments involving SARS-CoV-2 were performed in BSL-3. The plates were incubated further for 1 h; the fluids were discarded; the infected cells were rinsed with sterile PBS before adding with 1.5% carboxymethyl cellulose (CMC) (Sigma Aldrich, St. Louis, MO, United States) in complete DMEM and the plates were incubated further for 3 days (SARS-CoV-2) or 7 days (DENV, ZIKV, and JEV). After incubations, the infected cells were fixed with 10% formaldehyde at room temperature for 1 h (2 h for SARS-CoV-2). The cells were washed with distilled water five times to get rid of the CMC and stained with 1% crystal violet in 10% ethanol at room temperature for 15 min. After washing with distilled water, the plates were dried, and plaques were counted visually. The amount of the virus in the original sample was calculated: PFU/mL = plaque number/(infection volume × dilution factor).

The RD cells were seeded on a 24-well culture plate (1.5 × 10^5^ cells per well) and incubated at 37°C in a 5% CO_2_ incubator overnight. After discarding the supernatant, the 10-fold serially diluted supernatants of the superantibody-mediated virus replication inhibition experiments were added into appropriate RD cell-containing wells, and the plates were incubated further for 1 h. The fluids were removed and 1.5% CMC in complete DMEM was added to each well and incubated further for 72 h. The cells were fixed with formalin and stained with crystal violet dye as described earlier. Plaque number were counted by eyes, and the number of viruses in the original sample was calculated.

For PEDV, after incubating with 10-fold diluted samples, the extracellular fluids were discarded; the cells were rinsed with sterile PBS, added with 1.5% CMC (Sigma Aldrich, St. Louis, MO, United States) in DMEM containing N-tosyl-L-phenylalanyl chloromethyl ketone (TPCK) trypsin, and incubated at 37°C in a 5% CO_2_ atmosphere for 2 days. The cells were fixed with formalin and stained with crystal violet dye as described earlier. The CPE (syncytial formation) was enumerated under a microscope (40 × magnification), and the number of viruses in the original preparation was calculated.

### Focus-Forming Assay

Vero E6 cells (4 × 10^4^ cells/well) were seeded to wells of a 96-well cell culture plate and incubated at 37°C, 5% CO_2_ for 24 h. The cell-seeded plates were moved to the BSL-3 room. The samples containing viruses (SARS-CoV-2 omicron variant) were 10-fold serially diluted. The fluids were removed from the cell-containing wells and replaced with 50 μL of the diluted virus samples. After 1 h incubation, the fluids were removed, replaced with 1.5% CMC in complete DMEM, and incubated further at 37°C, 5% CO_2_ for 3 days. The CMC was removed from wells; the cells were fixed with 10% formaldehyde at room temperature for 2 h, washed with PBS three times, and permeated with 0.1% Triton X-100 in PBS at room temperature for 20 min. After washing two times with PBS, the cells were blocked with 5% BSA in PBS and stained with mouse anti-SARS-CoV-2 nucleoprotein antibody (1:5000) at room temperature for 1 h followed by incubating with goat anti-mouse IgG-HRP conjugate (SouthernBiotech, Birmingham, AL, United States). After the 1-h incubation, the cells were washed with PBS and the foci were developed by adding TMB sure blue substrate (SeraCare Life Sciences, Milford, MA, United States). The focal numbers were counted under an inverted microscope (40 × magnification). The numbers of foci (infectious virus particles) were calculated: FFU/mL = foci number/(infection volume × dilution factor).

For enumeration of HCV infectious particles, Huh7 cells (4 × 10^4^ cells/well) were seeded to wells of a 96-well cell culture plate and incubated at 37°C, 5% CO_2_ for 24 h. The fluids in all wells were discarded, and the cells were added with virus samples (culture supernatants from the superantibody-mediated inhibition of virus replication experiments) for 1 h; the fluids were discarded, the complete DMEM were replenished, and the infected cells were incubated for 3 days. The cells were fixed with 4% paraformaldehyde at room temperature for 20 min, washed, permeated with 0.1% Triton X-100, and blocked with 5% BSA in PBS. After blocking, the cells were probed with mouse anti-NS5A (Glab-ampai et al., 2017) for 1 h, washed, and added with goat anti-mouse Ig-alkaline phosphatase conjugate (Southern Biotech) for 1 h. After washing, the BCIP/NBT substrate (SeraCare Life Science) was added for color development. Numbers of foci were counted under an inverted light microscope, and the FFU/mL was calculated as mentioned earlier.

### Computerized Simulation to Determine Presumptive Interaction Between the Viral RNA-Dependent RNA Polymerase and the Human Single-Chain Antibody Variable Fragment

Amino acid sequences of the HuscFv34 and three-dimensional (3D) structures of RdRp of DENV serotypes 1 and 4 and of PEDV were submitted for protein modeling using AlphaFold2 (Jumper et al., 2021) available in ColabFold’s online notebook (Mirdita et al., 2022). Modeled 3D structure of the HuscFv34 was docked against existing crystal structures of RdRp of different viruses, [HCV (PDB ID: 1QUV), DENV serotype 2 (PDB ID: 6IZY), DENV serotype 3 (PDB ID: 2J7U), ZIKV (PDB ID: 6LD1), JEV (PDB ID: 4MTP), EV71 (PDB ID: 3N6L), CVA16 (PDB ID: 5Y6Z), and SARS-CoV-2 (PDB ID: 6M71)], and the predicted 3D structures of RdRp of DENV serotypes 1 and 4 and PEDV, *via* HADDOCK server version 2.4 (van Zundert et al., 2016). The parameters from the HADDOCK (HADDOCK scores, van der Waals energy, electrostatic energy, desolvation energy, restraint violation energy, buried surface area, and Z-Score) were collected. The intermolecular docking that showed the best HADDOCK score was selected. Pymol software (The PyMOL Molecular Graphics System, Version 2.5.2, Schrodinger, LLC, NY, United States) was used for building the molecular interactive protein structure models. The docked structures were further submitted to the PRODIGY server to predict the binding energy [ΔG (kcal per mol)] and K_*d*_ (M) at 25°C (Honorato et al., 2021).

### Statistical Analysis

GraphPad Prism version 9 software (GraphPad Software, San Diego, CA, United States^1^) was used for the calculation of the half-maximal effective dose (EC_50_) of the superantibody. Mean values and standard deviations (SD) of each treatment group from three independent experiments were compared using a one-way ANOVA. *P*-values of 0.05 or lower were considered statistically different: *p* > 0.05 (ns, not significant); *p* ≤ 0.05 (*), *p* ≤ 0.01 (^**^), *p* ≤ 0.001 (^***^), and *p* ≤ 0.0001 (^****^).

## Results

### Penetratin-Linked HuscFv34 to Hepatitis C Virus RNA-Dependent RNA Polymerase

**FIGURE 1:**
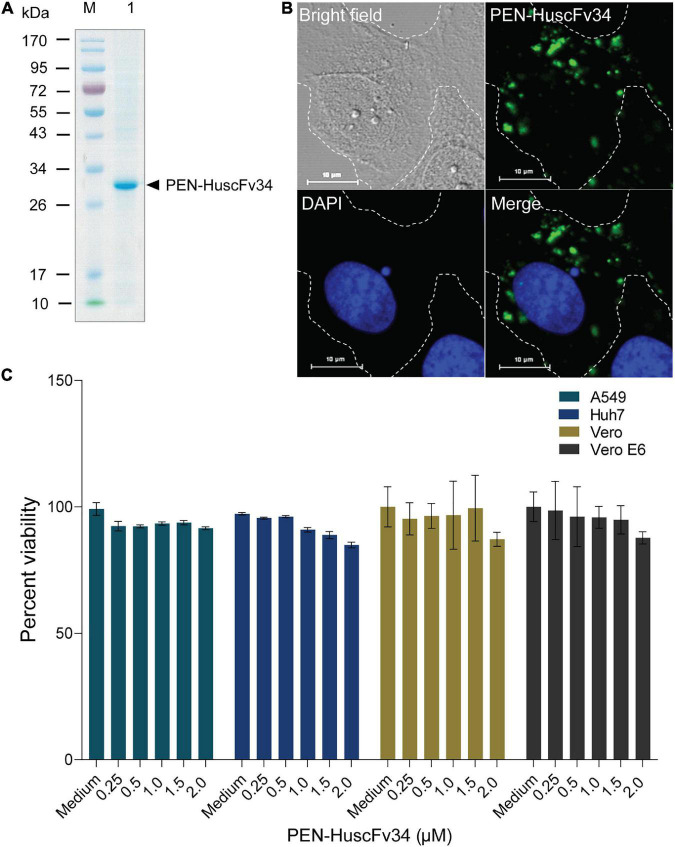
Characteristics of the penetratin (PEN)-HuscFv34 to HCV RdRp. **(A)** Purified PEN-HuscFv34 after SDS-PAGE and CBB staining. Lane M, Protein molecular mass standard; Lane 1, SDS-PAGE-separated purified PEN-HuscFv34 stained by CBB dye (∼30 kDa; arrowhead). **(B)** Cell-penetrating ability of the PEN-HuscFv34. The intracellular PEN-HuscFv34 stained green while nuclei are blue. **(C)** Biocompatibility of the PEN-HuscFv34 with mammalian cells including A549, Huh7, Vero, and Vero E6 cells. The PEN-HuscFv34 (superantibody) at the concentrations that were tested (0.25–2.0 μM) did not cause cytotoxicity to the cells. Percent viability of the cells (mean ± standard deviation) was not different from each other (*p* > 0.05).

Penetratin-linked human single-chain antibody variable fragments (PEN-HuscFv34) was produced from transformed BL21(DE3) *E. coli* grown under an IPTG-induced condition. The yield of the bacterial inclusion body obtained from the *E. coli* homogenate was 7.029 g/L of the bacterial culture. After the solubilization and refolding, the total protein obtained from each mg of the inclusion body was 680 μg. The CBB-stained SDS-PAGE-separated purified preparation revealed a single protein band with a relative mass of 30 kDa (Figure 1A), the correct molecular mass for PEN-HuscFv, in which each molecule consists of a VH domain linked to the VL domain *via* the (Gly_4_Ser)_3_ linker plus 16 amino acids (RQIKIWFQNRRMKWKK) of penetratin (Poungpair et al., 2010). As shown in Figure 1B, the PEN-HuscFv34 (green) could enter the mammalian cells (being cell-penetrable antibody/superantibody). The superantibody did not cause cytotoxicity to the mammalian cells that were tested, as determined by using the CytoTox-Glo™ Cytotoxicity Assay, based on the Practical Guide to ISO 109903-5 (Wallin, 1998; Figure 1C). The superantibody-treated cells appeared unchanged in their morphology under the light microscope (data not shown).

### Inhibition of RNA Virus Replication by Superantibody

**FIGURE 2:**
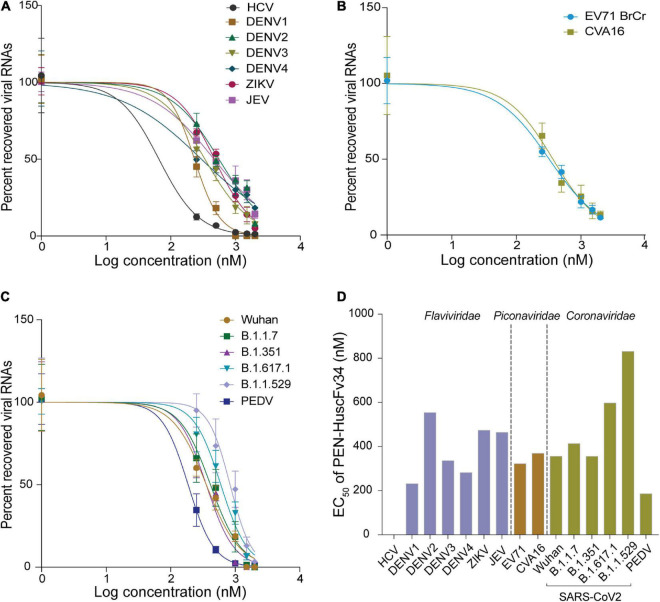
Inhibition of the plus-sense RNA virus replication by the superantibody shown as percent recovered viral RNAs inside the superantibody-treated infected cells, when compared with the infected cells in the medium alone. (A) Viruses of the family *Flaviviridae* (HCV, DENV1–4, ZIKV, and JEV); (B) Enteroviruses of the family *Picornaviridae* (EV71 and CVA16). (C) Members of the family *Coronaviridae* (genus *Betacoronavirus*: SARS-CoV-2 Wuhan wild-type and variants of concerns: α, β, δ, and omicron; and genus *Alphacoronavirus*: PEDV). (D) Half-maximal effective dose (EC_50_) of the superantibody against the tested viruses.

**FIGURE 3:**
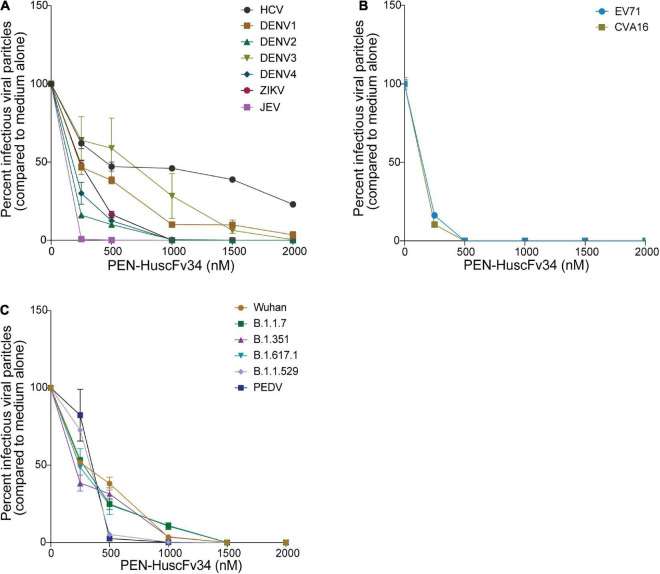
Reduction of the infectious viral particles released from the infected cells after treatment with various concentrations of the superantibody when compared with infected cells in the medium alone. (A) Viruses of the family *Flaviviridae*; (B) Enteroviruses of the family *Picornaviridae*; (C) Viruses of the family *Coronaviridae*.

The ability of the superantibody specific to HCV RdRp (PEN-HuscFv34) in inhibiting replication of the homologous virus and other plus-sense RNA viruses in the family *Flaviviridae* (DENV1-4, ZIKV, and JEV), *Picornaviridae* (EV71 and CVA16), and *Coronaviridae* (PEDV and SARS-CoV-2) was determined. Cells infected with the respective viruses were treated with different concentrations (0.25−2.0 μM) of the HCV-RdRp-specific superantibody or medium alone (negative control); the treated cells were subjected to real-time RT-PCR for quantification of the intracellular viral mRNAs, and their respective culture fluids were tested by PFA/FFA for enumeration of the released infectious particles. The superantibody could inhibit replication of the viruses that were tested in a dose-dependent manner as indicated by the percent reduction of the viral RNAs inside the infected cells compared to negative replication inhibition (medium) (Figures 2A–C). The superantibody also mediated the reduction of the numbers of the infectious particles released into the cell culture supernatants (Figures 3A–C). The EC_50_ of the superantibody on individual studied viruses is summarized in Figure 2D and Table 1. In the experiments, positive inhibition controls for individual viruses were not included as there are no approved direct-acting drugs/agents for certain viruses: DENV, ZIKV, JEV, EV71, CVA16, and PEDV. Details of the fold reduction of virus RNA from infected cells treated with the medium containing different concentrations of superantibody when compared with infected cells treated with the medium alone are shown in Supplementary Figures 1–3, and details of the reduction of released infectious viral particles (PFU/mL or FFU/mL) from the virus-infected cells treated with the medium-containing different concentrations of superantibody to RdRp when compared with infected cells treated with the medium alone are shown in Supplementary Figures 4–6.

**TABLE 1:** EC_50_ (nM) of penetratin (PEN)-HuscFv34 in replication inhibition of the tested viruses.

***Flaviviridae***							
Virus name	HCV	DENV1	DENV2	DENV3	DENV4	ZIKV	JEV
EC_50_	65.6	232	553.6	336.3	282.5	473.9	464.4
***Picornaviridae***							
Virus name	EV71	CVA16					
EC_50_	322.4	369.6					
***Coronaviridae***							
*Betacoronavirus*						*Alphacoronavirus*	
SARS-CoV-2						PEDV	
Variant	Wuhan	α (B.1.1.7)	β (B.1.351)	δ (B.1.617.1)	omicron (B.1.1.529)	GII	
EC_50_	356.4	413.4	355.7	597.7	831.6	186.3	

### Computerized Simulation to Determine Presumptive Interaction Between the Viral RNA-Dependent RNA Polymerase and the HuscFv34

**FIGURE 4:**
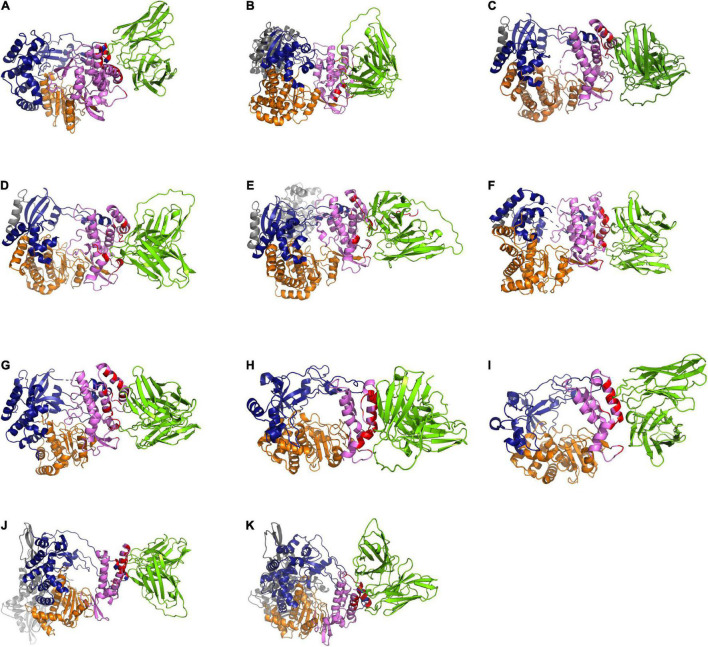
Computerized models of interaction between HuscFv34 and viral RdRp. (A–G) Viruses of the family *Flaviviridae* (HCV, DENV1–4, ZIKV, and JEV); (H,I) viruses of the family *Picornaviridae* (EV71 and CVA16); and (J,K) viruses of the family *Coronaviridae* (SARS-CoV-2 and PEDV). The RdRp are shown as cartoons: fingers domains (deep blue), palm domains (orange), and thumb domains (pink). The cartoon colored in red represents the contact interface between the HuscFv34 (green cartoon structure) and the target RdRp. Gray cartoons in DENV, ZIKV, and JEV are N-terminal *S*-adenosyl methionine methyltransferases (MTases). Gray cartoons in SARS-CoV-2 are the beta-hairpin that sandwiches with the palm domain, the Nidovirus-specific extension domain (NIRAN) domain, and the interface subdomain of the viral nsp12.

In this study, the *in-silico* interactions of the homology modeled HuscFv34 3D structure with RdRp of the HCV and other plus-sense RNA viruses were determined by using the available crystal structures of HCV, DENV serotypes 2 and 3, ZIKV, JEV, EV71, CVA16, and SARS-CoV-2 and modeled 3D structures of DENV serotypes 1 and 4 and PEDV, for which the crystal structures were not yet available. The data for computerized prediction of HuscFv34 and RdRp models and their interaction are summarized in Supplementary Table 2. The computerized models of interaction between the HuscFv34 and the RdRp of the studied viruses are shown in Figure 4. The details on the residues and domains of the RdRp of the viruses that formed contact interface with residues in the CDRs of the HuscFv34 are given in Table 2.

**TABLE 2:** Residues and domains of the RdRp of the viruses that formed contact interface with the residues in CDRs of the HuscFv34.

HCV RdRp		HuscFv34		Interactive bond
Residue	Region	Residue	Region	
A25	Finger	V167	VL-CDR1	Alkyl
N28	Finger	Q164	VL-CDR1	Hydrogen
N28	Finger	G165	VL-CDR1	Hydrogen
S29	Finger	H168	VL-CDR1	Hydrogen
S29	Finger	H169	VL-CDR1	Hydrogen
R32	Finger	Q235	VL-CDR3	Hydrogen
R32	Finger	S137	VH-CDR3	Hydrogen
R32	Finger	P237	VL-CDR3	Hydrogen
R32	Finger	N138	VH-CDR3	Hydrogen
S431	Thumb	H169	VL-CDR1	Hydrogen
R490	Thumb	Q62	VH-CDR2	Hydrogen
R498	Thumb	N57	VH-CDR2	Hydrogen
R498	Thumb	T58	VH-CDR2	Hydrogen
V499	Thumb	F236	VL-CDR3	Pi-Alkyl
H502	Thumb	D33	VH-CDR1	Hydrogen
H502	Thumb	W50	VH-CDR2	Pi-Pi
R503	Thumb	D103	VH-CDR3	Hydrogen
R503	Thumb	H169	VL-CDR1	Pi-Alkyl
R503	Thumb	T234	VL-CDR3	Hydrogen
K531	Thumb	N54	VH-CDR2	Hydrogen

**DENV1 RdRp**		**HuscFv34**		**Interactive bond**
**Residue**	**Region**	**Residue**	**Region**	

H800*	Thumb	G165	VL-CDR1	Hydrogen
T805*	Thumb	F236	VL-CDR3	Pi-Sigma
E806*	Thumb	Q164	VL-CDR1	Hydrogen
D807*	Thumb	H169	VL-CDR1	Hydrogen
D807*	Thumb	H168	VL-CDR1	Hydrogen
L809*	Thumb	H169	VL-CDR1	Hydrogen
S810	Thumb	V167	VL-CDR1	Hydrogen
S810	Thumb	H169	VL-CDR1	Hydrogen
S810	Thumb	H168	VL-CDR1	Hydrogen
R814	Thumb	G165	VL-CDR1	Hydrogen
V829	Thumb	H169	VL-CDR1	Pi-Anion
S830	Thumb	H169	VL-CDR1	Pi-Anion
S892	Thumb	N54	VH-CDR2	Electrostatic
D893	Thumb	S55	VH-CDR2	Electrostatic
L898	Thumb	D103	VH-CDR3	Hydrogen
W899	Thumb	D103	VH-CDR3	Hydrogen

**DENV2 RdRp**		**HuscFv34**		**Interactive bond**
**Residue**	**Region**	**Residue**	**Region**	

K719	Thumb	Q235	VL-CDR3	Hydrogen
R770	Thumb	H169	VL-CDR1	Hydrogen
E834	Thumb	H168	VL-CDR1	Salt bridge, Electrostatic
E834	Thumb	H169	VL-CDR1	Hydrogen
E834	Thumb	R238	VL-CDR3	Electrostatic
Y838	Thumb	H169	VL-CDR1	Electrostatic
R856	Thumb	H169	VL-CDR1	Hydrogen
R856	Thumb	Y102	VH-CDR3	Pi-Alkyl
A860	Thumb	Y102	VH-CDR3	Hydrogen
K861	Thumb	G105	VH-CDR3	Hydrogen
K861	Thumb	D106	VH-CDR3	Salt bridge, Electrostatic
N868	Thumb	N54	VH-CDR2	Hydrogen
D881	Thumb	N54	VH-CDR2	Hydrogen
D881	Thumb	S55	VH-CDR2	Hydrogen
D881	Thumb	N57	VH-CDR2	Hydrogen

**DENV3 RdRp**		**HuscFv34**		**Interactive bond**
**Residue**	**Region**	**Residue**	**Region**	

T806*	Thumb	H169	VL-CDR1	Hydrogen
E807*	Thumb	H169	VL-CDR1	Hydrogen
D808*	Thumb	H169	VL-CDR1	Hydrogen, Electrostatic
D808*	Thumb	Y102	VH-CDR3	Hydrogen
T832	Thumb	Y104	VH-CDR3	Hydrogen
W833	Thumb	Y104	VH-CDR3	Hydrogen
E834	Thumb	Y104	VH-CDR3	Hydrogen, Pi-Anion
E834	Thumb	S31	VH-CDR1	Hydrogen
E834	Thumb	H32	VH-CDR1	Electrostatic, Hydrogen
A860	Thumb	S55	VH-CDR2	Hydrogen
Q861	Thumb	R72	VH-CDR2	Hydrogen
L864	Thumb	N57	VH-CDR2	Hydrogen
E878	Thumb	Q164	VL-CDR1	Hydrogen
E878	Thumb	Q235	VL-CDR3	Hydrogen
L880	Thumb	H168	VL-CDR1	Pi-Sigma
D881	Thumb	F236	VL-CDR3	Pi-Anion, Pi-Sigma
Y882	Thumb	H169	VL-CDR1	Hydrogen
M883	Thumb	N52	VH-CDR2	Hydrogen

**DENV4 RdRp**		**HuscFv34**		**Interactive bond**
**Residue**	**Region**	**Residue**	**Region**	

K812	Thumb	D106	VH-CDR3	Hydrogen, Electrostatic
K812	Thumb	E108	VH-CDR3	Hydrogen, Electrostatic
P830	Thumb	T28	VH-CDR1	Hydrogen
H832	Thumb	H32	VH-CDR1	Hydrogen
H832	Thumb	G26	Electrostatic	
H832	Thumb	T28	VH-CDR1	Hydrogen, Electrostatic
E835	Thumb	Y104	VH-CDR3	Hydrogen
D836	Thumb	T30	VH-CDR1	Hydrogen
R872	Thumb	N170	VL-CDR1	Hydrogen
Y880	Thumb	N172	VL-CDR1	Hydrogen
D882	Thumb	N172	VL-CDR1	Hydrogen
P885	Thumb	Y102	VH-CDR3	Pi-Alkyl
R888	Thumb	N52	VH-CDR2	Hydrogen
E895	Thumb	H168	VL-CDR1	Hydrogen
E895	Thumb	Q235	VL-CDR3	Hydrogen
E895	Thumb	F236	VL-CDR3	Hydrogen
Y890	Thumb	Y102	VH-CDR3	Hydrogen
A892	Thumb	Y102	VH-CDR3	Pi-Alkyl

**ZIKV RdRp**		**HuscFv34**		**Interactive bond**
**Residue**	**Region**	**Residue**	**Region**	

K721	Thumb	H169	VL-CDR1	Hydrogen
L776	Thumb	G171	VL-CDR1	Hydrogen
K843	Thumb	N170	VL-CDR1	Pi-Cation
K843	Thumb	Q192	VL-CDR2	Hydrogen
G854	Thumb	Y174	VL-CDR1	Hydrogen
A862	Thumb	Y102	VH-CDR3	Hydrogen
A862, E863	Thumb	Y102	VH-CDR3	Pi-Alkyl
E863	Thumb	F236	VL-CDR3	Hydrogen
E863	Thumb	V167	VL-CDR1	Pi-Alkyl
E863	Thumb	S137	VH-CDR3	Hydrogen
I865	Thumb	D103	VH-CDR3	Amide-Pi Stacked
K866	Thumb	D103	VH-CDR3	Hydrogen, Alkyl
K866	Thumb	Y104	VH-CDR3	Hydrogen, Pi-Alkyl
K866	Thumb	G105	VH-CDR3	Hydrogen
K866	Thumb	Y107	VH-CDR3	Hydrogen
K866	Thumb	D33	VH-CDR1	Hydrogen
D884	Thumb	S55	VH-CDR2	Salt bridge, Electrostatic
D884	Thumb	N57	VH-CDR2	Electrostatic

**JEV RdRp**		**HuscFv34**		**Interactive bond**
**Residue**	**Region**	**Residue**	**Region**	

K724	Thumb	Q235	VL-CDR3	Hydrogen
K724	Thumb	G165	VL-CDR1	Hydrogen
K724	Thumb	V167	VL-CDR1	Hydrogen
R775	Thumb	N170	VL-CDR1	Hydrogen
T839	Thumb	H168	VL-CDR1	Hydrogen
T839	Thumb	H169	VL-CDR1	Hydrogen
D840	Thumb	H169	VL-CDR1	Hydrogen
Y843	Thumb	H169	VL-CDR1	Hydrogen
K846	Thumb	N170	VL-CDR1	Hydrogen
K846	Thumb	G171	VL-CDR1	Hydrogen
Y869	Thumb	Y104	VH-CDR3	Hydrogen
R876	Thumb	N54	VH-CDR2	Hydrogen
D886	Thumb	N54	VH-CDR2	Hydrogen
T889	Thumb	N57	VH-CDR2	Hydrogen
T889	Thumb	D103	VH-CDR3	Hydrogen
T889	Thumb	D33	VH-CDR1	Hydrogen

**EV71 RdRp**		**HuscFv34**		**Interactive bond**
**Residue**	**Region**	**Residue**	**Region**	

K427	Thumb	F236	VL-CDR3	Hydrogen
Q428	Thumb	Q164	VL-CDR1	Hydrogen
Q428	Thumb	G165	VL-CDR1	Hydrogen
Q428	Thumb	V167	VL-CDR1	Hydrogen
E431	Thumb	F236	VL-CDR3	Hydrogen
S435	Thumb	H168	VL-CDR1	Hydrogen
T436	Thumb	H169	VL-CDR1	Hydrogen
R438	Thumb	T58	VH-CDR2	Hydrogen
R444	Thumb	Y104	VH-CDR3	Pi-Cation
R444	Thumb	D33	VH-CDR1	Electrostatic
R444	Thumb	N57	VH-CDR2	Hydrogen
R444	Thumb	D103	VH-CDR3	Hydrogen, Electrostatic
L446	Thumb	N57	VH-CDR2	Hydrogen

**CVA16 RdRp**		**HuscFv34**		**Interactive bond**
**Residue**	**Region**	**Residue**	**Region**	

H383	Thumb	N57	VH-CDR2	Pi-Sigma
H383	Thumb	T58	VH-CDR2	Hydrogen
H383	Thumb	G59	VH-CDR2	Hydrogen
Q384	Thumb	N54	VH-CDR2	Hydrogen
K427	Thumb	D33	VH-CDR1	Electrostatic
K427	Thumb	F236	VL-CDR3	Pi-Anion
E428	Thumb	P237	VL-CDR3	Hydrogen
E428	Thumb	Y102	VH-CDR3	Hydrogen
E428	Thumb	R238	VL-CDR3	Electrostatic
E431	Thumb	H169	VL-CDR1	Salt bridge, Electrostatic
E431	Thumb	H169	VL-CDR1	Pi-Alkyl
K432	Thumb	Q164	VL-CDR1	Hydrogen
V434	Thumb	H169	VL-CDR1	Hydrogen
S435	Thumb	F236	VL-CDR3	Pi-Alkyl
R438	Thumb	H169	VL-CDR1	Hydrogen
R438	Thumb	H168	VL-CDR1	Salt bridge, Electrostatic
N450	Thumb	D103	VH-CDR3	Salt bridge, Electrostatic
N450	Thumb	Q235	VL-CDR3	Hydrogen

**PEDV RdRp**		**HuscFv34**		**Interactive bond**
**Residue**	**Region**	**Residue**	**Region**	

K412	Fingers	H169	VL-CDR1	Hydrogen
E413	Fingers	H169	VL-CDR1	Electrostatic, Hydrogen
E413	Fingers	Y102	VH-CDR3	Hydrogen

**PEDV RdRp**		**HuscFv34**		**Interactive bond**
**Residue**	**Region**	**Residue**	**Region**	

E420	Fingers	H169	VL-CDR1	Electrostatic, Hydrogen
E420	Fingers	Q235	VL-CDR3	Hydrogen
E420	Fingers	H168	VL-CDR1	Salt bridge, Electrostatic, Hydrogen
H885	Thumb	D103	VH-CDR3	Pi-Anion, Hydrogen
K888	Thumb	D103	VH-CDR3	Salt bridge, Electrostatic
K888	Thumb	D33	VH-CDR1	Electrostatic
K888	Thumb	W50	Pi-Cation	
K888	Thumb	Y104	VH-CDR3	Pi-Alkyl
N891	Thumb	Y104	VH-CDR3	Hydrogen
A892	Thumb	Y104	VH-CDR3	Pi-Alkyl
E896	Thumb	D106	VH-CDR3	Hydrogen
E896	Thumb	H32	VH-CDR1	Electrostatic

**SARS-CoV-2 RdRp**		**HuscFv34**		**Interactive bond**
**Residue**	**Region**	**Residue**	**Region**	

F415	Fingers	N54	VH-CDR2	Hydrogen
N416	Fingers	S55	VH-CDR2	Hydrogen
D418	Fingers	S55	VH-CDR2	Hydrogen
D418	Fingers	N52	VH-CDR2	Hydrogen
D418	Fingers	Y104	VH-CDR3	Hydrogen
K426	Fingers	Y102	VH-CDR3	Hydrogen
K849	Thumb	S55	VH-CDR2	Hydrogen
Q886	Thumb	H169	VL-CDR1	Hydrogen
R889	Thumb	H169	VL-CDR1	Hydrogen, Pi-Alkyl
K890	Thumb	D103	VH-CDR3	Salt bridge, Electrostatic
K890	Thumb	H169	VL-CDR1	Pi-Alkyl
D893	Thumb	H168	VL-CDR1	Salt bridge, Electrostatic
E894	Thumb	N57	VH-CDR2	Hydrogen
E894	Thumb	F236	VL-CDR3	Pi-Sigma

*Priming loop of the thumb domain.

## Discussion

RNA-dependent RNA polymerase (RdRp) is an inscribed protein of RNA viruses that is indispensable for the virus replication cycle. The protein is a principal component of the replicase/transcriptase complex that generates new genomic RNA and virus proteins which assemble to form virus progeny for further spread. RdRp is structurally conserved among the RNA viruses with no human homolog. Therefore, it is a potential target for a pan-anti-RNA virus agent. Currently, several small chemical inhibitors (both nucleoside and non-nucleoside inhibitors) that target the RdRp have been developed and tested for the treatment of the RNA virus infections; some of them have been approved and launched for clinical use while the others are at various phases of clinical trials or were discontinued (Tian et al., 2021). Examples of the nucleoside inhibitors that target RdRp are sofosbuvir (Sovaldi/PSI-7977/GS-7977) for the treatment of hepatitis B and C, favipiravir (T-705/Avigan/Favipiravir, Favilavir) for the treatment of influenza (repurposed for COVID-19 treatment), ribavirin (ICN-1229/Tribavirin) for the treatment of influenza, hepatitis C, and respiratory syncytial virus (RSV) infection (repurposed for the treatment of SARS in 2003 and COVID-19), and remdesivir for COVID-19 and other infections. More recently, a few non-nucleoside inhibitors of RdRp have been launched for the treatment of hepatitis C including dasabuvir (Exviera/Viekira Pak/Viekira XR/ABT-333) and lomibuvir (VX-222/VCH-222) (Tian et al., 2021). Limitations of the chemical inhibitors besides their off-target and adverse side effects such as teratogenicity, hemolytic anemia, gastro-intestinal disturbance, and others that preclude patients’ compliance are their susceptibility to virus mutation; thus, often they must be used in combined medication among themselves, with other drugs or an interferon, for the treatment of viruses of the drug-resistant phenotypes, such as genotype I HCV.

Antibodies have been used for the treatment of human diseases including infectious, non-infectious, and toxin/venom-mediated maladies. For safety issues, the therapeutic antibodies or antibodies for passive immunization should have negligible immunogenicity in the recipients, implying that the fully human isotype is the safest antibody format. Although the penetratin (PEN) that has been linked to the HuscFv34 is derived from the third helix of *Drosophila* Antennapedia homeodomain protein (Derossi et al., 1994), it has been shown that dendritic cells (DCs) pulsed with this peptide could not activate autologous T cells, implying that the peptide is not immunogenic (Brooks et al., 2015). Currently, the PEN has been used in several vaccine studies to deliver tumor-associated antigens into antigen-presenting cells (APCs), and as a non-viral gene delivery vehicle in DNA vaccines, as well as carrying therapeutic substances into cellular compartments (reviewed by Brooks et al., 2010; Yang et al., 2019). However, in preclinical and clinical trials, the immunogenicity and biocompatibility of the PEN-HuscFvs must be investigated.

Antibody uses several residues in multiple CDRs in synergistic binding to the target, causing difficulty for the pathogens to create an antibody-escape target mutant that retains the inherent functional activity, particularly the proteins that require high conservation. The main concern in using therapeutic antibodies in the treatment of the virus infection is the antibody-dependent enhancement (ADE) (Kulkarni, 2020) that often aggravates the morbidity. Conventional antibodies elicit ADE by different mechanisms. For *Flavivirus* infection, the Fc fragments of the virus-antibody complexes bind to the Fc-receptors and enhance the virus entry to myeloid cells, leading to increment of the virus replication and viral load (extrinsic ADE) (Khandia et al., 2018). The intracellular virus may inhibit type 1 interferon response and activates the production of interleukin-10 that causes a type 2 (Th2) immune response bias, which heightens virus production and release (intrinsic ADE); the intrinsic ADE enhanced more DENV replication than the extrinsic ADE (Narayan and Tripathi, 2020). For other viruses, including respiratory viruses such as RSV, influenza virus, and coronavirus, the bi-/multi-valent antibodies may form large immune complexes that activate complement, causing the release/formation of anaphylatoxins, chemotaxis, and membrane attack complexes (MAC) that recruit immune and inflammatory cells to the infected areas and exacerbate the tissue inflammation, cytokine storm, cellular apoptosis, and multi-organ damage, i.e., the so-called immune enhancement ADE (Sánchez-Zuno et al., 2021). The antibody may promote virus entry to host cells by other mechanisms besides the Fc-mediated; for SARS-CoV-2, non-neutralizing antibodies to an epitope in the N-terminal domain (NTD) of the S1 subunit of the spike protein promote an upstanding/open form of the RBD by cross-linking two adjacent spike trimers, which then enhances the virus entry (Liu et al., 2021). For the influenza virus, the non-neutralizing antibody promotes the virus entry by increasing hemagglutinin stem flexibility and virus fusion to the cell membrane (Winarski et al., 2019). The antibodies may enhance entry of SARS-CoV-2 into monocytes/macrophages *via* the Fc receptors; nevertheless, the infection is abortive; instead, the virus induces a specific M2 macrophage transcriptional program and causes host immune paralysis for the benefit of COVID-19 progression and pathogenesis (Boumaza et al., 2021). In this study, the superantibody (PEN-HuscFv34) specific to intracellular RdRp that works inside the cells cannot bind to the Fc receptors on cells and cannot form large immune complexes (cannot activate complement) but inhibited the replication of RNA viruses across families, is offered for testing further as a safe and broadly effective anti-RNA virus agent. Usually, the superantibodies (the term coined by Charles Morgan, president of InNexus Biotechnology, Vancouver, WA, Canada) enter cells; if there is no target, they leave the cells and enter new cells. The superantibodies bind intracellular targets and eventually the antibody-bound substances are eliminated by the normal cell physiological process, including the ubiquitin-proteasome and/or autophagy. “The beauty of the sole human antibodies is that they have minimal, if there were any, immunogenicity; thus, they should be less or not toxic. Besides, they are highly discriminating, i.e., far more specific than small-molecule drugs” (Coghlan, 2022). They are more tolerable to target mutation than the small chemical drugs as they bind to several target sites by using many residues in multiple CDRs.

The HCV RdRp epitope bound by the HuscFv34 was identified previously (by phage mimotope search using 12-mer peptide phage display library and competitive peptide ELISA) as a conformational epitope that is composed of residues in the finger’s tip of the finger domain and helix O of the thumb domain of the HCV RdRp (NS5B protein), which were juxtaposed upon the protein folding to form the roof of the active enzymatic groove (closed catalytic tunnel) (Thueng-In et al., 2014). There were three phage mimotopic peptides (mimotopes 1−3; M1-M3) derived from the mimotope search that matched with the stretched sequence of the HCV RdRp, including M1: ALPFMGYHNSVY matched with 22PISPLSNSLLRHHNLVY40 of the Δ1 loop of finger domain; M2: NYPATNTHRYTP matched with residues 470GLSAFTLHSYFT481; and M3: IPVKSWPIRPSS matched with residues 495PPLRAWRHRARA506 of the thumb domain (based on the identical, conserved, and semiconserved amino acid residues upon the pairwise alignment) (Thueng-In et al., 2014). In this study, the computerized simulation of the HuscFv34-HCV RdRp interaction was performed to verify the results of the previous finding. We did not model the interaction of the superantibody (PEN-HuscFv) with the target RdRps because the penetratin (PEN) was linked to the HuscFv by a flexible linker and another end of the PEN was free. Besides, the PEN itself is not structured. Therefore, it should be inappropriate to fix the PEN in the rigid model for modeling and intermolecular docking as in reality the PEN would move freely while the HuscFv would be the principal part involved in target binding. By the *in-silico* analysis, the HuscFv34 interacted with residues of the HCV RdRp fingers domain, i.e., A25, N28, S29, and R32, located at the finger’s Δ1 extension loop [residues I11-S46)] that usually packs against the thumb domain to form active closing (form 1) of the HCV RdRp channel (Bressanelli et al., 1999). Binding of the HuscFv34 at the finger’s Δ1 extension loop could disturb the conformation and rigidity of the enzymatic groove (Biswal et al., 2005). Besides the fingertip, the HuscFv34 also formed a contact interface with many residues at the back of the thumb domain. Previous evidence has shown that interaction of the HCV NS5B (RdRp) with a host component, nucleolin, is indispensable for HCV replication (Shimakami et al., 2006). Residue W500 and three arginines (R498, R501, and R503) at the armadillo-like arm repeats of the thumb domain (Bressanelli et al., 1999) are important for the nucleolin binding and the HCV replication (Kusakawa et al., 2007). The HuscFv34 interaction with several residues in this region of the thumb domain (shown in Figure 4A and Table 2) may interfere with the RdRp-host nucleolin interaction, hence HCV replication inhibition.

For dengue viruses, the RdRp is located at the C-terminal residues 270 to 900 of the bifunctional NS5 protein that contains 900 amino acids (the N-terminal residues of the NS5 form the enzyme S-adenosyl methionine transferase) (Yap et al., 2007). The thumb domain (residues 706−900) of the RdRp contains a motif (motif E/primer grip) that lies between the palm domain and α-helices of the thumb domain (Yap et al., 2007). There is a loop that spans amino acids 782 to 809 of the thumb domain, called a priming loop. The priming loop together with another loop of the finger domain form the roof of the tunnel that regulates RNA entry and exit from the RdRp active site (Yap et al., 2007). Several residues of the priming loop protrude into the RdRp active groove and stabilize the NTPs on the RNA template at the initial stage of the *de novo* RNA synthesis; these residues also pad alongside the RNA template during the process of the RNA synthesis (Yap et al., 2007; Gong and Peersen, 2010). The thumb domain is also involved in the motility of the newly synthesized RNA. Various interactive bonds (hydrogen, salt bridge, stacking interaction) between amino acid residues of the priming loop, including Thr794 and Ser796, Glu807 and Arg815, and Arg749 and Trp787, contribute to maintaining the orientation of the RdRp protein (Yap et al., 2007). From the *in-silico* prediction, the HuscFv34 interacted with several residues in the priming loops of DENV1 and DENV3 of the thumb domain (asterisks in Table 2) that may interfere with their functional activity and/or cause a structural change of the protein, leading to impairment of the RdRp activity, hence the DENV replication inhibition.

From the *in-silico* analysis, the HuscFv34 is also predicted to form interaction with many residues at the back surface of the thumb domains of DENV1, DENV3, PEDV, and SARS-CoV-2 and interacted solely with the C-terminal helices of thumb domains of DENV2, DENV4, ZIKV, JEV, EV71, and CVA16, which could be the site of the polymerase interaction with other viral/host cellular proteins during the formation of the replication/transcriptase complex and replication initiation (Bressanelli et al., 1999). Several non-nucleoside chemical inhibitors have been shown to bind to allosteric sites on the outer surface of the thumb subdomain (Thumb II or T2) and cause changes in the spatial conformation of the enzyme, rendering it inactive and reducing the viral load (Le Pogam et al., 2006; De Clercq, 2013; Li et al., 2016; Lim et al., 2016; Tian et al., 2021).

The EC_50_ of the superantibody (PEN-HuscFv34) was found in the nanomolar range for all of the tested RNA viruses, ranging from 65.6 nM for the homologous HCV to 831.6 for SARS-CoV-2 omicron variant, which was comparable to the chemical nucleoside and non-nucleoside inhibitors: favipiravir EC_50_ for SARS-CoV-2 was 61.88 μM (Wang et al., 2020); cytosine analog (NHC, EIDD-1931) EC_50_ values for SARS-CoV-2 and MERS-CoV were 0.3 and 0.56 μM, respectively (Sheahan et al., 2020); EC_50_ values of remdesivir (GS-5734) in inhibiting SARS-CoV and MERS-CoV in human airway epithelial cells (HAE) were 0.069 and 0.07/0.074 μM, respectively (Sheahan et al., 2017; Agostini et al., 2018), and SARS-CoV-2 in Vero E6 cells were 0.77 μM (Wang et al., 2020) and 23.15 μM (Choy et al., 2020); EC_50_ value of ribavirin in inhibiting SARS-CoV-2 in Vero E6 cells was 109.5 μM (Wang et al., 2020; Frediansyah et al., 2021).

## Conclusion

The cell-penetrating human single-chain antibody variable fragments (superantibody) specific to NS5B (RdRp) of HCV that were found previously to inhibit the HCV replication and that rescued the HCV suppressed host innate (anti-viral) immunity were tested against other RNA viruses of the families *Flaviviridae* (DENV1-4, ZIKV, JEV), *Picornaviridae* (EV71 and CVA16), and *Coronaviridae* (genus *Alphacoronavirus*: PEDV and genus *Betacoronavirus*: SARS-CoV-2 including Wuhan wild type and variants of concerns including alpha, beta, delta, and omicron). The superantibody inhibited replication of all RNA viruses that were tested in a dose-dependent manner. *In-silico* analysis indicated that the superantibody interacted mainly with the armadillo-like arm repeats at the back of the RdRp thumb domain, which may cause allosterical changes in the spatial conformation of the RdRp, rendering the enzyme inactive, hence virus replication inhibition. Although the molecular mechanisms of the superantibody against the viruses await experimental elucidation, data of this study persuade testing the superantibody further toward clinical application as a pan-direct acting anti-RNA virus agent.

## Data Availability Statement

The original contributions presented in the study are included in the article/Supplementary Material, further inquiries can be directed to the corresponding author.

## Author Contributions

WC, MC, and KG-A contributed to the conceptualization, funding acquisition, resources, and project administration. MC, NS, and WC contributed to the methodology, data curation, formal analysis, supervision, visualization, and writing and editing the manuscript. KG-A, TT, TS, WT, KM, and MC contributed to the investigation, methodology, and visualization. KK and MC did the computerization. All authors have read and agreed to the published version of the manuscript.

## Conflict of Interest

The authors declare that the research was conducted in the absence of any commercial or financial relationships that could be construed as a potential conflict of interest.

## Publisher’s Note

All claims expressed in this article are solely those of the authors and do not necessarily represent those of their affiliated organizations, or those of the publisher, the editors and the reviewers. Any product that may be evaluated in this article, or claim that may be made by its manufacturer, is not guaranteed or endorsed by the publisher.

## Footnotes

1
www.graphpad.com

## References

[B1] Abbass S. Kamal E. Salama M. Salman T. Sabry A. Abdel-Razek W. (2021). Efficacy and safety of sofosbuvir plus daclatasvir or ravidasvir in patients with COVID-19: a randomized controlled trial. *J. Med. Virol.* 93 6750–6759. 10.1002/jmv.27264 34379337 PMC8426808

[B2] Agostini M. L. Andres E. L. Sims A. C. Graham R. L. Sheahan T. P. Lu X. (2018). Coronavirus susceptibility to the antiviral remdesivir (GS-5734) is mediated by the viral polymerase and the proofreading exoribonuclease. *mBio* 9 e00221–e00218. 10.1128/mBio.00221-18 29511076 PMC5844999

[B3] Biswal B. K. Cherney M. M. Wang M. Chan L. Yannopoulos C. G. Bilimoria D. (2005). Crystal structures of the RNA-dependent RNA polymerase genotype 2a of hepatitis C virus reveal two conformations and suggest mechanisms of inhibition by non-nucleoside inhibitors. *J. Biol. Chem.* 280 18202–18210. 10.1074/jbc.M413410200 15746101

[B4] Boumaza A. Gay L. Mezouar S. Bestion E. Diallo A. B. Michel M. (2021). Monocytes and macrophages, targets of severe acute respiratory syndrome coronavirus 2: the clue for coronavirus disease 2019 immunoparalysis. *J. Infect. Dis.* 224, 395–406. 10.1093/infdis/jiab044 33493287 PMC7928817

[B5] Bressanelli S. Tomei L. Roussel A. Incitti I. Vitale R. L. Mathieu M. (1999). Crystal structure of the RNA-dependent RNA polymerase of hepatitis C virus. *Proc. Natl. Acad. Sci. U. S. A.* 96 13034–13039. 10.1073/pnas.96.23.13034 10557268 PMC23895

[B6] Brooks N. A. Esparon S. Pouniotis D. Pietersz G. A. (2015). Comparative immunogenicity of a cytotoxic T cell epitope delivered by penetratin and TAT cell penetrating peptides. *Molecules* 20 14033–14050. 10.3390/molecules200814033 26247926 PMC6332296

[B7] Brooks N. A. Pouniotis D. S. Tang C. K. Apostolopoulos V. Pietersz G. A. (2010). Cell-penetrating peptides: application in vaccine delivery. *Biochim. Biophys. Acta* 1805 25–34. 10.1016/j.bbcan.2009.09.004 19782720

[B8] Bruenn J. A. (2003). A structural and primary sequence comparison of the viral RNA-dependent RNA polymerases. *Nucleic Acids Res.* 31 1821–1829. 10.1093/nar/gkg277 12654997 PMC152793

[B9] Chan-Yeng M. Xu R. H. Shi Z. (2015). Bat origin of human coronaviruses. *Virol. J.* 12:21. 26689940 10.1186/s12985-015-0422-1 PMC4687304

[B10] Choy K. T. Wong A. Y. Kaewpreedee P. Sia S. F. Chen D. Hui K. P. Y. (2020). Remdesivir, lopinavir, emetine, and homoharringtonine inhibit SARS-CoV-2 replication in vitro. *Antiviral Res.* 178:104786. 10.1016/j.antiviral.2020.104786 32251767 PMC7127386

[B11] Coghlan A. (2022). *Super Antibodies Break the Cell Barrier.* London, UK: NewScientist.

[B12] De Clercq E. (2013). Antivirals: past, present and future. *Biochem. Pharmacol.* 85 727–744. 10.1016/j.bcp.2012.12.011 23270991

[B13] Derossi D. Joliot A. H. Chassaing G. Prochiantz A. (1994). The third helix of the Antennapedia homeodomain translocates through biological membranes. *J. Biol. Chem.* 269 10444–10450. 8144628

[B14] European Centre for Disease Prevention and Control (2020). *Dengue Worldwide Overview.* Solna: European Centre for Disease Prevention and Control.

[B15] Frediansyah A. Nainu F. Dhama K. Mudatsir M. Harapan H. (2021). Remdesivir and its antiviral activity against COVID-19: a systematic review. *Clin. Epidemiol. Glob. Health* 9 123–127. 10.1016/j.cegh.2020.07.011 32838064 PMC7410793

[B16] Glab-ampai K. Chulanetra M. Malik A. A. Juntadech T. Thanongsaksrikul J. Srimanote P. (2017). Human single chain-transbodies that bound to domain-I of non-structural protein 5A (NS5A) of hepatitis C virus. *Sci. Rep.* 7:15042. 10.1038/s41598-017-14886-9 29118372 PMC5678119

[B17] Gong P. Peersen O. B. (2010). Structural basis for active site closure by the poliovirus RNA-dependent RNA polymerase. *Proc. Natl. Acad. Sci. U. S. A.* 107 22505–22510. 10.1073/pnas.1007626107 21148772 PMC3012486

[B18] Gorbalenya A. E. Pringle F. M. Zeddam J. L. Luke B. T. Cameron C. E. Kalmakoff J. (2002). The palm subdomain-based active site is internally permuted in viral RNA-dependent RNA polymerases of an ancient lineage. *J. Mol. Biol.* 324 47–62. 10.1016/s0022-2836(02)01033-1 12421558 PMC7127740

[B19] Honorato R. V. Koukos P. I. Jimenez-Garcia B. Tsaregorodtsev A. Verlato M. Giachetti A. (2021). Structural biology in the clouds: the WeNMR-EOSC Ecosystem. *Front. Mol. Biosci.* 8:729513. 10.3389/fmolb.2021.729513 34395534 PMC8356364

[B20] Hu B. Ge X. Wang L. F. Shi Z. (2015). Bat origin of human coronaviruses. *Virol. J.* 12:221. 26689940 10.1186/s12985-015-0422-1 PMC4687304

[B21] Jia H. Gong P. (2019). A structure-function diversity survey of the RNA-dependent RNA polymerases from the positive-strand RNA viruses. *Front. Microbiol.* 10:1945. 10.3389/fmicb.2019.01945 31507560 PMC6713929

[B22] Jumper J. Evans R. Pritzel A. Green T. Figurnov M. Ronneberger O. (2021). Highly accurate protein structure prediction with AlphaFold. *Nature* 596 583–589. 10.1038/s41586-021-03819-2 34265844 PMC8371605

[B23] Jung K. Hu H. Saif L. J. (2016). Porcine deltacoronavirus infection: etiology, cell culture for virus isolation and propagation, molecular epidemiology and pathogenesis. *Virus Res.* 226 50–59. 10.1016/j.virusres.2016.04.009 27086031 PMC7114557

[B24] Khandia R. Munjal A. Dhama K. Karthik K. Tiwari R. Malik Y. S. (2018). Modulation of Dengue/Zika virus pathogenicity by antibody-dependent enhancement and strategies to protect against enhancement in Zika virus infection. *Front. Immunol.* 9:597. 10.3389/fimmu.2018.00597 29740424 PMC5925603

[B25] Kulkarni R. (2020). “Antibody-dependent enhancement of viral infections,” in *Dynamics of Immune Activation in Viral Diseases*, ed. Bramhachari P. V. (Singapore: Springer Singapore), 9–41.

[B26] Kulkeaw K. Sakolvaree Y. Srimanote P. Tongtawe P. Maneewatch S. Sookrung N. (2009). Human monoclonal ScFv neutralize lethal Thai cobra, *Naja kaouthia*, neurotoxin. *J. Proteomics* 72 270–282. 10.1016/j.jprot.2008.12.007 19162253

[B27] Kusakawa T. Shimakami T. Kaneko S. Yoshioka K. Murakami S. (2007). Functional interaction of hepatitis C virus NS5B with nucleolin GAR domain. *J. Biochem.* 141 917–927. 10.1093/jb/mvm102 17569707

[B28] Kyle J. L. Harris E. (2008). Global spread and persistence of dengue. *Ann. Rev. Microbiol.* 62 71–92. 18429680 10.1146/annurev.micro.62.081307.163005

[B29] Le Pogam S. Kang H. Harris S. F. Leveque V. Giannetti A. M. Ali S. (2006). Selection and characterization of replicon variants dually resistant to thumb- and palm-binding nonnucleoside polymerase inhibitors of the hepatitis C virus. *J. Virol.* 80 6146–6154. 10.1128/JVI.02628-05 16731953 PMC1472602

[B30] Li J. Johnson K. A. (2016). Thumb site 2 inhibitors of hepatitis C viral RNA-dependent RNA polymerase allosterically block the transition from initiation to elongation. *J. Biol. Chem.* 291, 10067–10077. 10.1074/jbc.M115.708354 26851276 PMC4858960

[B31] Lim S. P. Noble C. G. She C. C. Soh T. S. El Sahili A. Chan G. K. (2016). Potent allosteric dengue virus NS5 polymerase inhibitors: mechanism of action and resistance profiling. *PLoS Pathog.* 12:e1005737. 10.1371/journal.ppat.1005737 27500641 PMC4976923

[B32] Liu Y. Soh W. T. Kishikawa J. I. Hirose M. Nakayama E. E. Li S. (2021). An infectivity-enhancing site on the SARS-CoV-2 spike protein targeted by antibodies. *Cell* 184 3452–3466.e18. 10.1016/j.cell.2021.05.032 34139176 PMC8142859

[B33] Mirdita M. Schütze K. Moriwaki Y. Heo L. Ovchinnikov S. Steinegger M. (2022). ColabFold: making protein folding accessible to all. *Nat. Methods* 19, 679–682. 10.1038/s41592-022-01488-1 35637307 PMC9184281

[B34] Narayan R. Tripathi S. (2020). Intrinsic ADE: the dark side of antibody dependent enhancement during dengue infection. *Front. Cell. Infect. Microbiol.* 10:580096. 10.3389/fcimb.2020.580096 33123500 PMC7573563

[B35] Noobrakhsh F. Abdolmohammadi K. Fatahi Y. Dalili H. Rasoolinejad M. Rezaei F. (2019). Zika virus infection, basic and clinical aspects: a review article. *Iran. J. Public Health* 48 20–31. 30847308 PMC6401583

[B36] Novel Influenza A/H1N1 Investigation Team (2009). Description of the early stage of pandemic (H1N1) 2009 in Germany, 27 April-16 June 2009. *Euro. Surveill.* 14:19295. 10.2807/ese.14.31.19295-en 19660249

[B37] Payne S. (2017). “Chapter 10—Introduction to RNA viruses,” in *Viruses*, ed. Payne S. (Cambridge, MA: Academic Press), 97–105.

[B38] Pensaert M. B. de Bouck P. (1978). A new coronavirus-like particle associated with diarrhea in swine. *Arch. Virol.* 58 243–247. 83132 10.1007/BF01317606 PMC7086830

[B39] Phanthong S. Densumite J. Seesuay W. Thanongsaksrikul J. Teimoori S. Sookrung N. (2020). Human antibodies to VP4 inhibit replication of enteroviruses across subgenotypes and serotypes and enhance host innate immunity. *Front. Microbiol.* 11:562768. 10.3389/fmicb.2020.562768 33101238 PMC7545151

[B40] Picarazzi F. Vicenti I. Saladini F. Zazzi M. Mori M. (2020). Targeting the RdRp of emerging RNA viruses: the structure-based drug design challenge. *Molecules* 25:5695. 10.3390/molecules25235695 33287144 PMC7730706

[B41] Poch O. Sauvaget I. Delarue M. Tordo N. (1989). Identification of four conserved motifs among the RNA-dependent polymerase encoding elements. *EMBO J.* 8 3867–74. 10.1002/j.1460-2075.1989.tb08565.x 2555175 PMC402075

[B42] Poungpair O. Pootong A. Maneewatch S. Srimanote P. Tongtawe P. Songserm T. (2010). A human single chain transbody specific to matrix protein (M1) interferes with the replication of influenza A virus. *Bioconjug. Chem.* 21 1134–1141. 10.1021/bc900251u 20560610

[B43] Sánchez-Zuno G. A. Matuz-Flores M. G. González-Estevez G. Nicoletti F. Turrubiates-Hernández F. J. Mangano K. (2021). A review: antibody-dependent enhancement in COVID-19: the not so friendly side of antibodies. *Int. J. Immunopathol. Pharmacol.* 35:20587384211050199. 10.1177/20587384211050199 34632844 PMC8512237

[B44] Schmidt N. J. Lennette E. H. Ho H. H. (1974). An apparently new enterovirus isolated from patients with disease of the central nervous system. *J. Infect. Dis.* 129 304–309. 4361245 10.1093/infdis/129.3.304

[B45] Sheahan T. P. Sims A. C. Graham R. L. Menachery V. D. Gralinski L. E. Case J. B. (2017). Broad-spectrum antiviral GS-5734 inhibits both epidemic and zoonotic coronaviruses. *Sci. Transl. Med.* 9:eaal3653. 10.1126/scitranslmed.aal3653 28659436 PMC5567817

[B46] Sheahan T. P. Sims A. C. Zhou S. Graham R. L. Pruijssers A. J. Agostini M. L. (2020). An orally bioavailable broad-spectrum antiviral inhibits SARS-CoV-2 in human airway epithelial cell cultures and multiple coronaviruses in mice. *Sci. Transl. Med.* 2:eabb5883. 10.1126/scitranslmed.abb5883 32253226 PMC7164393

[B47] Shimakami T. Honda M. Kusakawa T. Murata T. Shimotohno K. Kaneko S. (2006). Effect of hepatitis C virus (HCV) NS5B-nucleolin interaction on HCV replication with HCV subgenomic replicon. *J. Virol.* 80 3332–3340. 10.1128/JVI.80.7.3332-3340.2006 16537600 PMC1440399

[B48] Tang X. Tian G. Zhao J. Zhou K. Y. (1998). Isolation and characterization of prevalent strains of avian influenza viruses in China. *Chin. J. Anim. Poult. Infect. Dis.* 20 1–5.

[B49] te Velthuis A. J. (2014). Common and unique features of viral RNA-dependent polymerases. *Cell. Mol. Life. Sci.* 71, 4403–4420. 10.1007/s00018-014-1695-z 25080879 PMC4207942

[B50] Thavorasak T. Chulanetra M. Glab-ampai K. Teeranitayatarn K. Songserm T. Yodsheewan R. (2022). Novel neutralizing epitope of PEDV S1 protein identified by IgM monoclonal antibody. *Viruses* 14:125. 10.3390/v14010125 35062329 PMC8778753

[B51] Thueng-In K. Thanongsaksrikul J. Jittavisutthikul S. Seesuay W. Chulanetra M. Sakolvaree Y. (2014). Interference of HCV replication by cell penetrable human monoclonal scFv specific to NS5B polymerase. *mAbs* 6 1327–1339. 10.4161/mabs.29978 25517317 PMC4622650

[B52] Tian L. Qiang T. Liang C. Ren X. Jia M. Zhang J. (2021). RNA-dependent RNA polymerase (RdRp) inhibitors: the current landscape and repurposing for the COVID-19 pandemic. *Eur. J. Med. Chem.* 213:113201. 10.1016/j.ejmech.2021.113201 33524687 PMC7826122

[B53] van Zundert G. C. P. Rodrigues J. P. G. L. M. Trellet M. Schmitz C. Kastritis P. L. Karaca E. (2016). The HADDOCK2.2 Web Server: user-friendly integrative modeling of biomolecular complexes. *J. Mol. Biol.* 428 720–725. 10.1016/j.jmb.2015.09.014 26410586

[B54] Venkataraman S. Prasad B. V. L. S. Selvarajan R. (2018). RNA dependent RNA polymerases: insights from structure, function and evolution. *Viruses* 10:76. 10.3390/v10020076 29439438 PMC5850383

[B55] Wakita T. Pietschmann T. Kato T. Date T. Miyamoto M. Zhao Z. (2005). Production of infectious hepatitis C virus in tissue culture from a cloned viral genome. *Nat. Med.* 11 791–796. 10.1038/nm1268 15951748 PMC2918402

[B56] Wallin R. F. (1998). *A Practical Guide to ISO 109903-5: Cytotoxicity. Medical Devices and Diagnostic Industry*. Available online at: https://www.mddionline.com/testing/practical-guide-iso-10993-5-cytotoxicity (accessed July 9, 2021).

[B57] Wang M. Cao R. Zhang L. Yang X. Liu J. Xu M. (2020). Remdesivir and chloroquine effectively inhibit the recently emerged novel coronavirus (2019-nCoV) in vitro. *Cell Res.* 30 269–271. 10.1038/s41422-020-0282-0 32020029 PMC7054408

[B58] Winarski K. L. Tang J. Klenow L. Lee J. Coyle E. M. Manischewitz J. (2019). Antibody-dependent enhancement of influenza disease promoted by increase in hemagglutinin stem flexibility and virus fusion kinetics. *Proc. Natl. Acad. Sci. U. S. A.* 116 15194–15199. 10.1073/pnas.1821317116 31296560 PMC6660725

[B59] World Health Organization [WHO] (2004). *The 4th Edition of the Manual for the Virological Investigation of Polio.* Geneva: WHO.

[B60] World Health Organization [WHO] (2019). *Middle East Respiratory Syndrome Coronavirus (MERS-CoV).* Geneva: WHO.

[B61] World Health Organization Ebola Response Team (2014). Ebola virus disease in West Africa – the first 9 months of the epidemic and forward projections. *N. Engl. J. Med.* 371 1481–1495. 10.1056/NEJMoa1411100 25244186 PMC4235004

[B62] Wu J. Liu W. Gong P. (2015). A structural overview of RNA-dependent RNA polymerases from the Flaviviridae family. *Int. J. Mol. Sci.* 16 12943–12957. 10.3390/ijms160612943 26062131 PMC4490480

[B63] Yang J. Luo Y. Shibu M. A. Toth I. Skwarczynskia M. (2019). Cell-penetrating peptides: efficient vectors for vaccine delivery. *Curr. Drug. Deliv.* 16 430–443. 10.2174/1567201816666190123120915 30760185 PMC6637094

[B64] Yap T. L. Xu T. Chen Y. L. Malet H. Egloff M. P. Canard B. (2007). Crystal structure of the dengue virus RNA-dependent RNA polymerase catalytic domain at 1.85-angstrom resolution. *J. Virol.* 81 4753–4765. 10.1128/JVI.02283-06 17301146 PMC1900186

[B65] Zein A. F. M. Z. Sulistiyana C. S. Raffaello W. M. Wibowo A. Pranata R. (2021). Sofosbuvir with daclatasvir and the outcomes of patients with COVID-19: a systematic review and meta-analysis with GRADE assessment. *Postgrad. Med. J.* 21 1–6. 10.1136/postgradmedj-2021-140287 37066509

