# Supplementary material for: Targeting Emerging RNA Viruses by Engineered Human Superantibody to Hepatitis C Virus RNA-Dependent RNA Polymerase
Source: Front Microbiol. 2022 Jul 22;13:926929. doi: 10.3389/fmicb.2022.926929 (PMC9355540; doi:10.3389/fmicb.2022.926929)
Supplement: Supplementary file 1 [file Data_Sheet_1.docx]

**Supplementary Table 1 ⎮** Oligonucleotide primer sequences for real-time RT-PCR used in this study.

| **Virus** | **Primer name** | **Sequence (5′-3′)** |
| --- | --- | --- |
| HCV | UTRLC1 | CTTCACGCAGAAAGCGTCTA |
|  | UTRLC2 | CAAGCACCCTATCAGGCAGT |
| DENV1 serotype 1 | DENV1NS5 forward | CAAAAGGAAGTCGYGCAATA |
|  | DENV1NS5 reverse | CTGAGTGAATTCTCTCTGCTRAAC |
| DENV1 serotype 2 | DENV2E forward | CAGGCTATGGCACYGTCACGAT |
|  | DENV2E reverse | CCATYTGCAGCARCACCATCTC |
| DENV1 serotype 3 | DENV3prM forward | GGACTRGACACACGCACCCA |
|  | DENV3prM reverse | CATGTCTCTACCTTCTCGACTTGYCT |
| DENV1 serotype 4 | DENV4prM forward | TTGTCCTAATGATGCTRGTCG |
|  | DENV4prM reverse | TCCACCYGAGACTCCTTCCA |
| ZKIV | ZIKV forward | CAGCTGGCATCATGAAGAAYC |
|  | ZIKV reverse | CACYTGTCCCATCTTYTTCTCC |
| JEV | JEV forward | AGAGCGGGGAAAAAGGTCAT |
|  | JEV reverse | TTTCACGCTCTTTCTACAGT |
| EV71 | PAN-EV forward | CGCAAAGACTGAACCCACTAATTT |
|  | PAN-EV reverse | TTGCCTCTGTTGTTACTTGGAGAT |
| CVA16 | VP1 forward | ATGGKTATGYWAAYTGGGACAT |
|  | VP1 reverse | CCTGACRTGYTTMATCCTCAT |
| PEDV | PEDV-N forward | CAAGCACTTCTGTTTCCCCGG |
|  | PEDV-N reverse | ATTGTCACCATAAGCAGCCA |
| SARS-CoV-2 | SARS-CoV-2 E forward | ACAGGTACGTTAATAGTTAATAGCGT |
|  | SARS-CoV-2 E reverse | ATATTGCAGCAGTACGCACACA |
| RPLPO (endogenous control; gene for ribosomal protein lateral stalk subunit P0) | forward | AGATGCAGCAGATCCGCAT |
|  | reverse | GGATGGCCTTGCGCA |

**Supplementary Table 2 ⎮** Summary of the computerized homology modeling and molecular docking of HuscFv34 and viral RdRp 3D/crystal structures

| Virus | HCV | DENV1 | DENV2 | DENV3 | DENV4 | ZIKV | JEV | EV71 BrCr | CVA16 | PEDV | SARS-CoV-2 |
| --- | --- | --- | --- | --- | --- | --- | --- | --- | --- | --- | --- |
| **Parameters retrieved from HADDOCK** | | | | | | | | | | | |
| **HADDOCK score** | -97.9 ± 13.6 | -109.6 ± 10.4 | -90.5 ± 5.5 | -69.1 ± 16.5 | -127 ± 4.3 | -84.7 ± 8.1 | -66.7 ± 8.1 | -79.6 ± 8.0 | -100.8 ± 3.7 | -97.2 ± 12.8 | -85.5 ± 2.2 |
| **Van der Waals energy** | -72.9 ± 8.5 | -82.1 ± 8.4 | -59.6 ± 4.9 | -56.6 ± 1.5 | -68.1 ± 7.0 | -72.2 ± 6.0 | -53.2 ± 3.9 | -65.4 ± 5.5 | -64.3 ± 8.5 | -43.4 ± 5.1 | -47.8 ± 7.3 |
| **Electrostatic energy** | -168.9 ± 6.8 | -182.6 ± 26.2 | -269.1 ± 43.3 | -177.7 ± 16.6 | -384.2 ± 41.9 | -211.7 ± 17.7 | -159.4 ± 16.0 | -249.2 ± 35.9 | -354.1 ± 28.1 | -412.0 ± 39.5 | -329.6 ± 39.2 |
| **Desolvation energy** | -10.3 ± 1.7 | -22.1 ± 4.8 | -1.6 ± 1.1 | -16.4 ± 2.1 | 2.8 ± 1.7 | -2.4 ± 0.4 | -9.0 ± 0.7 | 6.6 ± 5.9 | 2.6 ± 1.3 | -3.9 ± 4.0 | -2.7 ± 1.4 |
| **Restraint violation energy** | 190.2 ± 55.8 | 311.1 ± 84.5 | 246.1 ± 63.5 | 395.5 ± 23.4 | 148.0 ± 39.8 | 321.7 ± 36.1 | 273.5 ± 71.9 | 290.2 ± 34.6 | 317.4 ± 75.2 | 324.5 ± 48.3 | 308.6 ± 45.6 |
| **Buried surface area** | 2124.8 ± 112.2 | 2306.8 ± 100.3 | 1867.1 ± 203.4 | 1796.7 ± 234.7 | 2286.2 ± 97.7 | 2138.9 ± 81.7 | 1655.6 ± 45.8 | 2026.5 ± 37.2 | 1817.5 ± 72.5 | 1778.4 ± 72.8 | 1557.3 ± 26.6 |
| **Z-Score** | -1.9 | -1.6 | -1.8 | 0.3 | -2.1 | -2.0 | -0.3 | -0.7 | -2.1 | -1.5 | -1.5 |
| **Parameters retrieved from PRODIGY** | | | | | | | | | | | |
| **ΔG (kcal mol^-1^)** | -11.0 | -11.9 | -11.5 | -12.7 | -13.0 | -11.9 | -9.2 | -11.6 | -8.2 | -10.4 | -9.3 |
| **K_d_ (M) at 25 ℃** | 8.8E-09 | 1.9E-09 | 3.9E-09 | 4.5E-10 | 2.9E-10 | 1.7E-09 | 1.7E-07 | 3.1E-09 | 8.9E-07 | 2..4E-08 | 1.4E-07 |


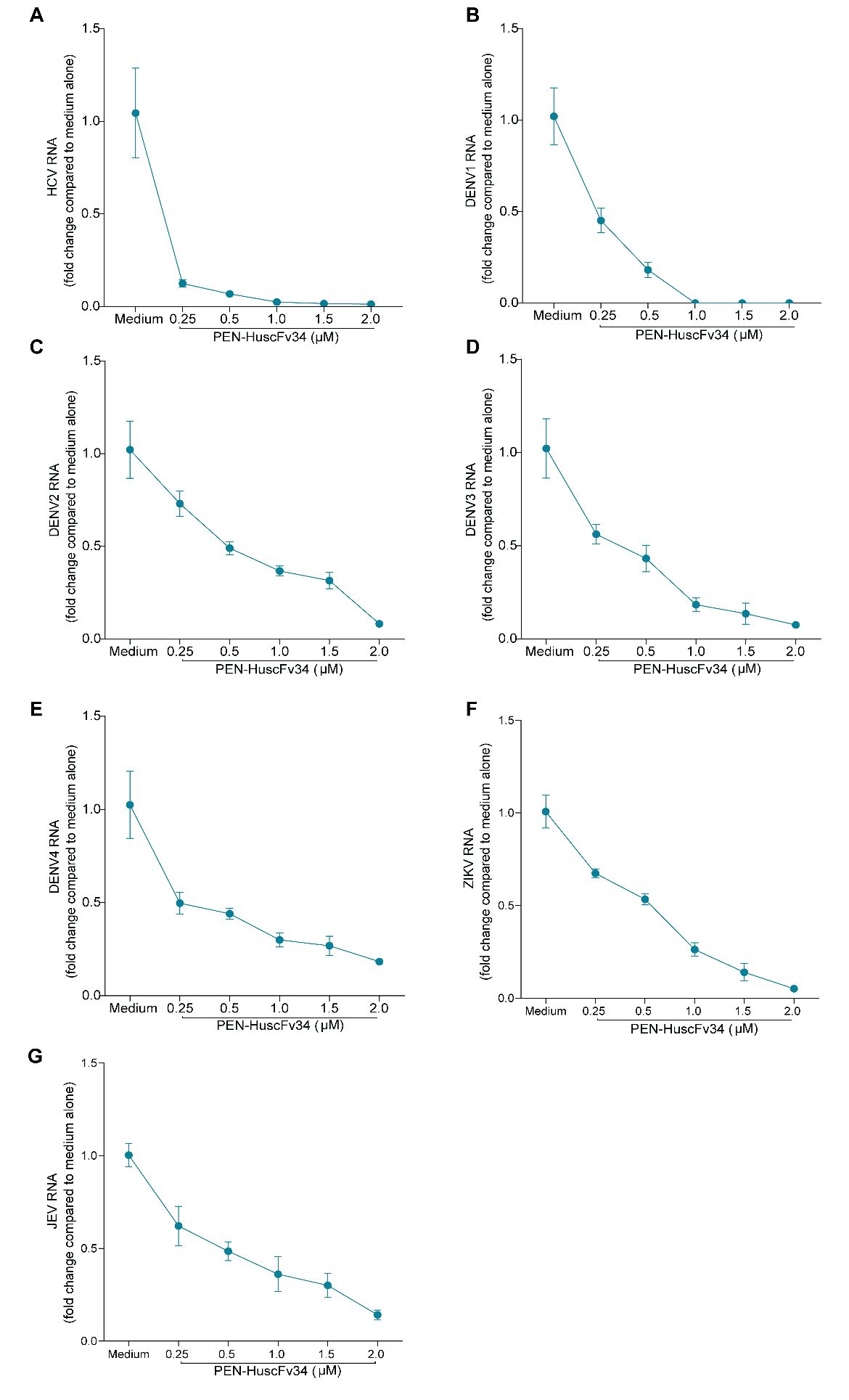


**Supplementary Figure 1 ⎮** Fold changes of virus RNAs from Flavivirus infected cells treated with medium containing different concentrations of superantibody, compared to infected cells treated with medium alone. (**A**) HCV, (**B-E**) DENV1-4, respectively, (**F**) ZIKV, and (**G**) JEV.


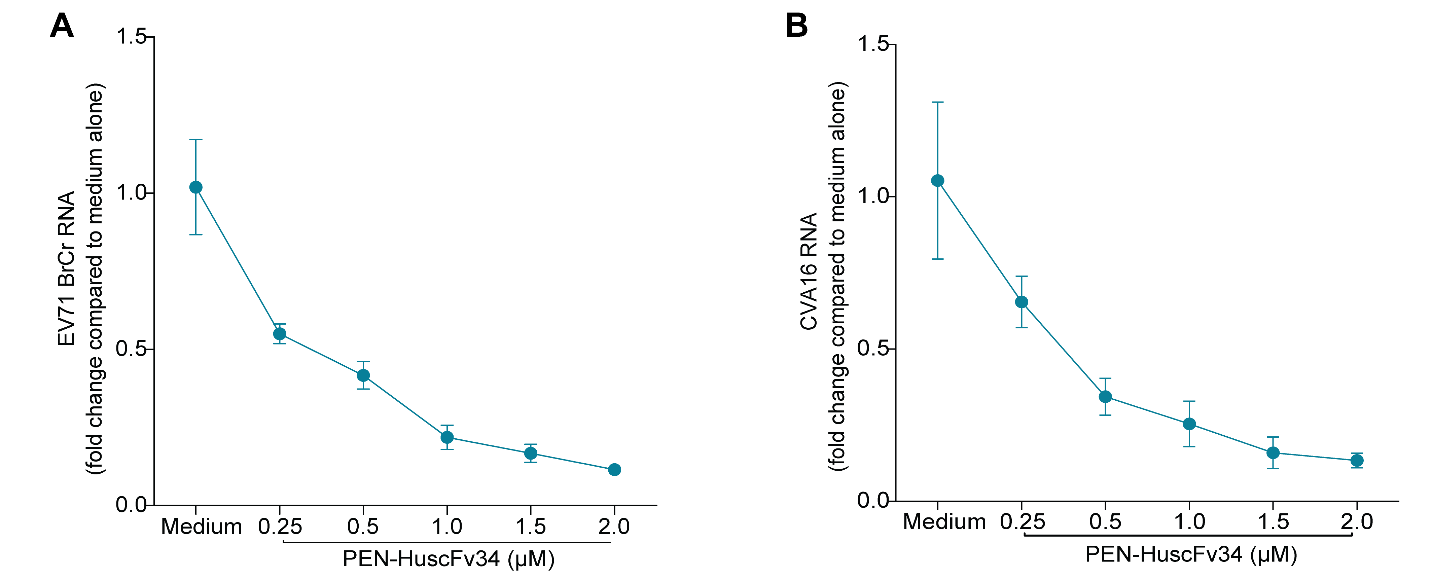


**Supplementary Figure 2 ⎮** Fold changes of virus RNAs from Picornavirus infected cells treated with medium containing different concentrations of superantibody, compared to infected cells treated with medium alone. (**A**) EV71 BrCr and (**B**) CVA16.


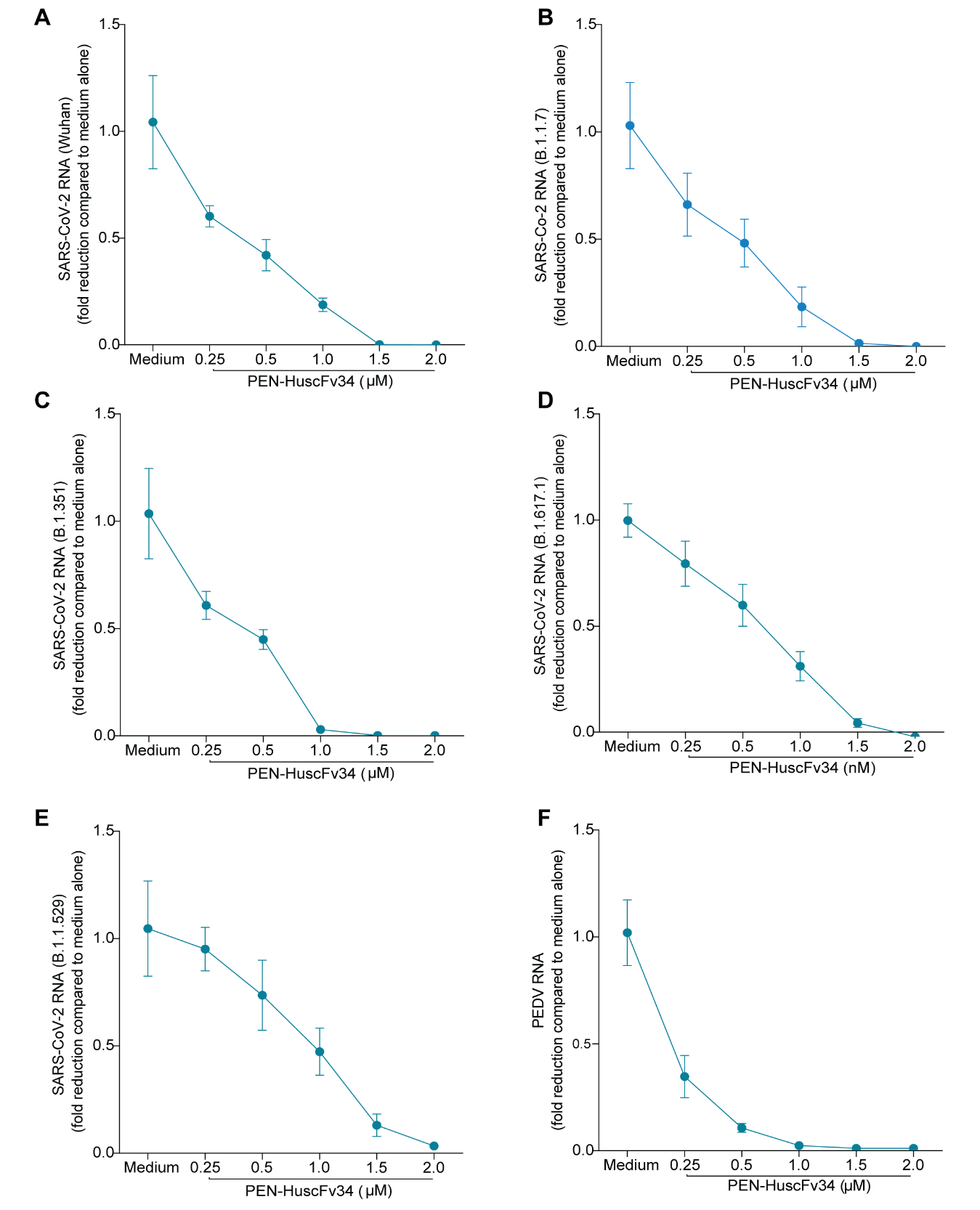


**Supplementary Figure 3⎮** Fold changes of virus RNAs from Coronavirus infected cells treated with medium containing different concentrations of superantibody, compared to infected cells treated with medium alone. (**A-E**) genus *Betacoronavirus*: SARS-CoV-2 Wuhan wild type and variants of concerns: α, β, δ and omicron; and (**F**) genus *Alphacoronavirus*: PEDV.


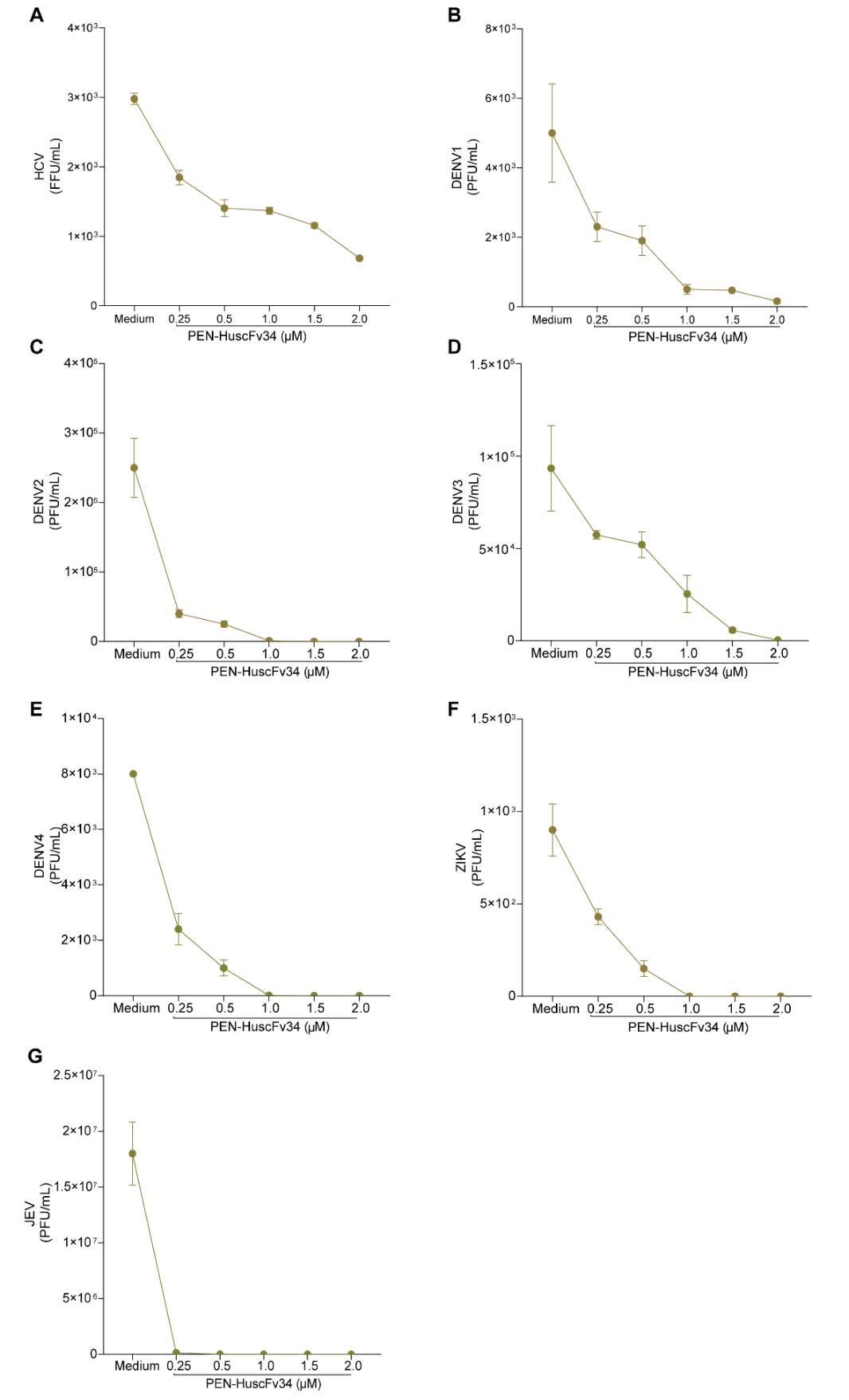


**Supplementary Figure 4 ⎮** Reduction of released infectious viral particles (FFU/mL or PFU/mL) from Flavivirus infected cells treated with medium containing different concentrations of superantibody to RdRp compared to infected cells treated with medium alone. (**A**) HCV, (**B-E**) DENV1-4, respectively, (**F**) ZIKV, and (**G**) JEV.


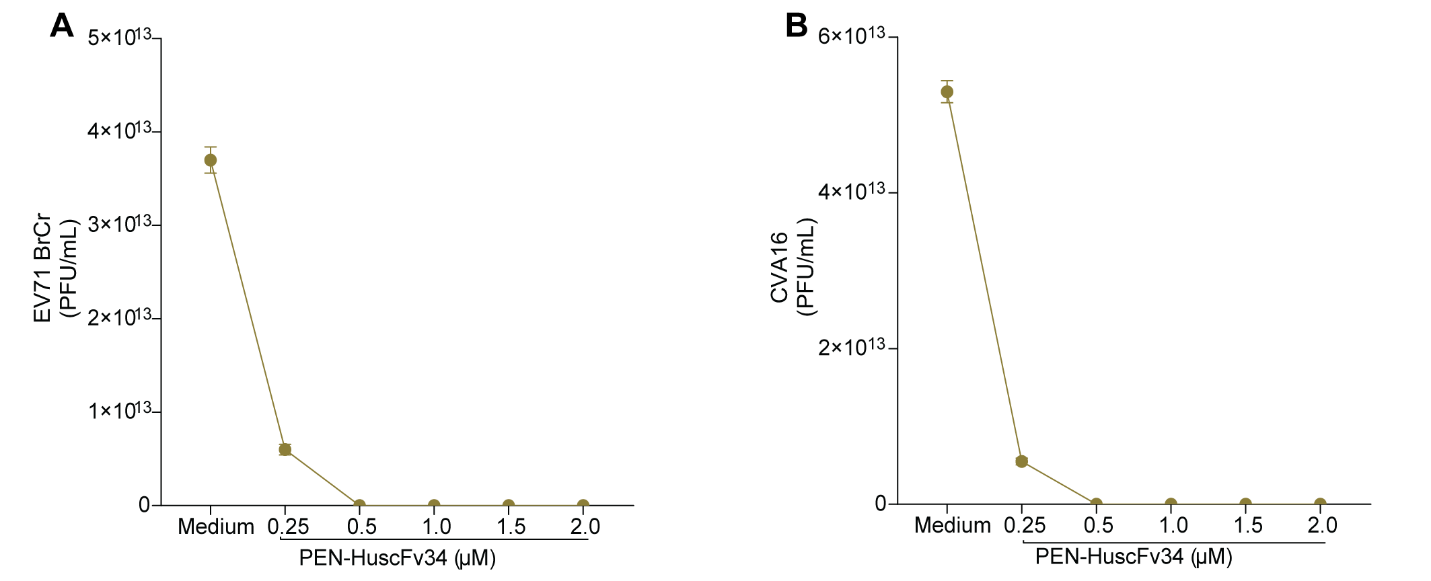


**Supplementary Figure 5 ⎮** Reduction of released infectious viral particles (PFU/mL) from Picornavirus infected cells treated with medium containing different concentrations of superantibody to RdRp compared to infected cells treated with medium alone. (**A**) EV71 BrCr and (**B**) CVA16.


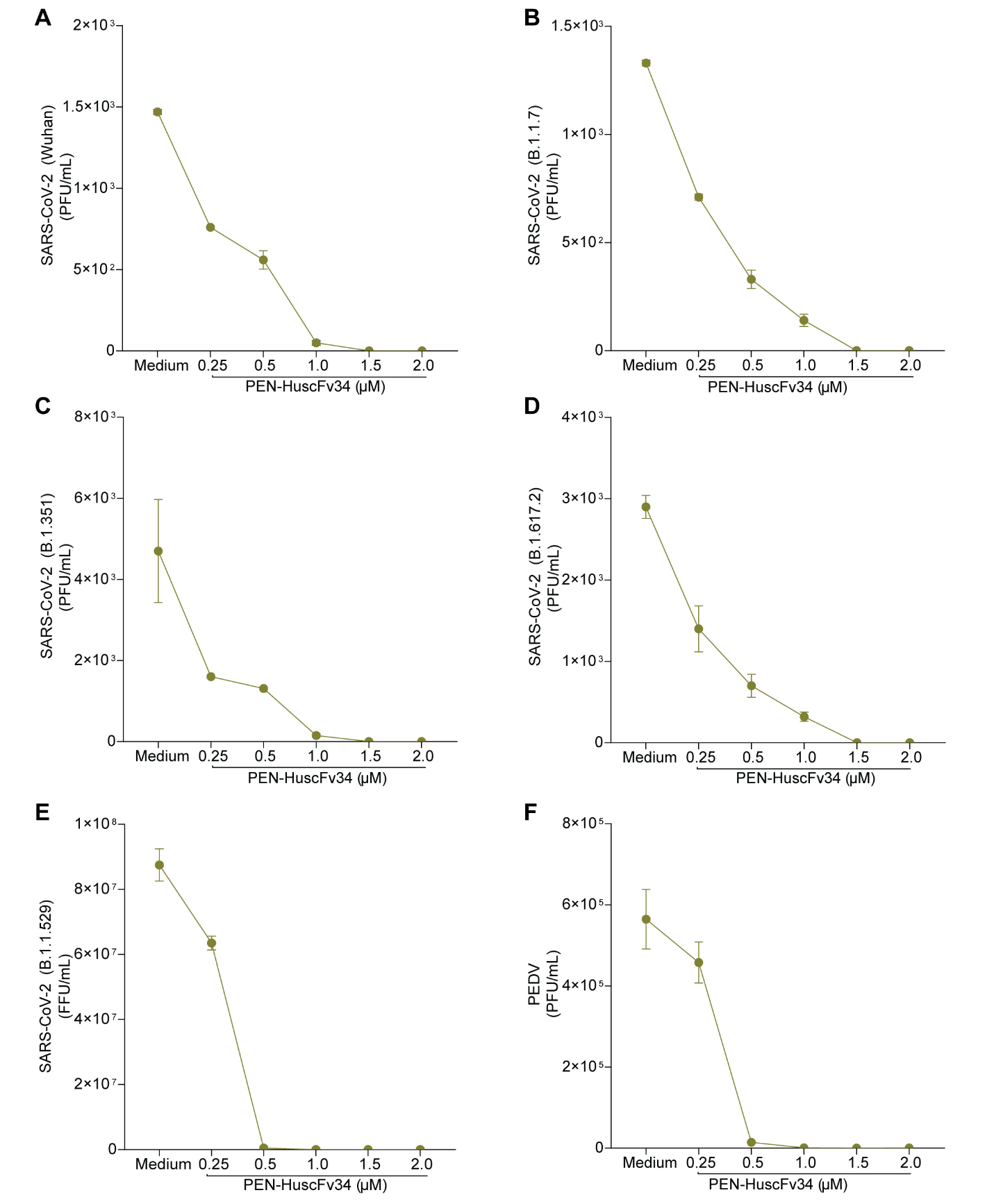


**Supplementary Figure 6 ⎮** Reduction of released infectious viral particles (FFU/mL or PFU/mL) from Coronavirus infected cells treated with medium containing different concentrations of superantibody to RdRp compared to variants of concerns: α, β, δ and omicron. (**F**) Genus *Alphacoronavirus*: PEDV.
